# Atmospheric Chemistry of Chloroprene Initiated by OH Radicals: Combined *Ab Initio*/DFT Calculations and Kinetics Analysis

**DOI:** 10.1021/acs.jpca.4c05428

**Published:** 2024-10-08

**Authors:** Parandaman Arathala, Rabi A. Musah

**Affiliations:** † Department of Chemistry, University at Albany—State University of New York, 1400 Washington Avenue, Albany, New York 12222, United States; ‡ Department of Chemistry, Louisiana State University, Baton Rouge, LA 12222, United States

## Abstract

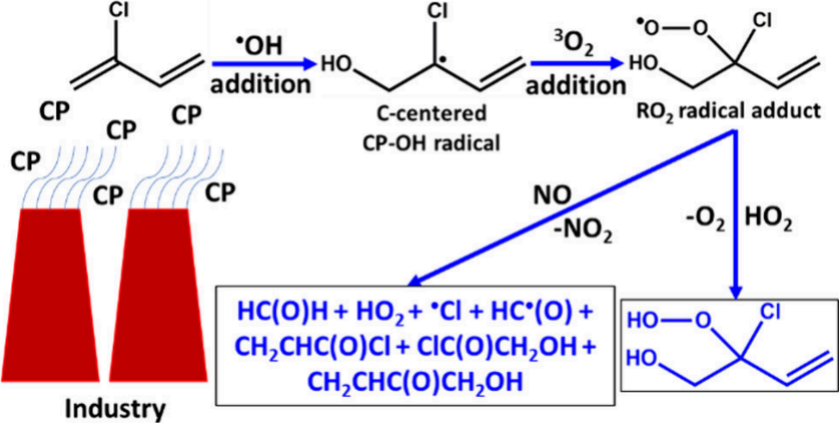

Chloroprene (CP; CH_2_=C(Cl)–CH=CH_2_) is a significant toxic airborne pollutant, often originating from anthropogenic activities. However, the environmental fate of CP is incompletely understood. High level CCSD(T)/aug-cc-pVTZ//M06-2X/aug-cc-pVTZ calculations combined with kinetic modeling were employed here to glean new insight into the reaction mechanism, energies, and kinetics of the reaction of CP with OH radical (^•^OH). We report the energies of four different addition pathways and six different abstraction pathways. The ^•^OH attack on the terminal C_1_ atom of the =CH_2_ group (which is directly attached to the =CCl moiety), leading to the formation of HOCH_2_–^•^C(Cl)–CH=CH_2_, was found to be a major path. The barrier height for the formation of the corresponding transition state was found to be −1.9 kcal mol^–1^ below that of the starting CP + ^•^OH reactants. Rate coefficients were calculated for addition and abstraction pathways involving the CP + ^•^OH reaction under pre-equilibrium approximation conditions, employing a combination of canonical variational transition state theory and small curvature tunneling. The overall rate coefficient for the reaction of CP + ^•^OH at 298 K was found to be 1.4 × 10^–10^ cm^3^ molecule^–1^ s^–1^. The thermochemistry of the possible channels and atmospheric implications are provided. In addition, the fate of HOCH_2_–^•^C(Cl)–CH=CH_2_ in the presence of ^3^O_2_ was investigated. We found the reaction of the CP-derived peroxy radical adduct with HO_2_ and NO to make contributions to the formation of products such as formaldehyde, HO_2_ radical, Cl atom, HOCH_2_C(OOH)(Cl)CH=CH_2_, HOCH_2_C(O)Cl, ClC(O)CH=CH_2_, HOCH_2_C(O)CH=CH_2_, and HC(O) radical.

## 1 Introduction

Regulatory bodies across various jurisdictions have been investigating methods to minimize the public’s exposure to hazardous airborne substances. However, significant quantities of anthropogenic organic compounds are still emitted into the troposphere, which raises substantial worries about their escalating threats to the environment and to human health.^1^ Among these air contaminants, the chlorinated volatile organic compounds (Cl-VOCs) are important in part because they are ubiquitous and emitted into the environment in enormous quantities.^2,3^ Many of them are harmful, carcinogenic, and hazardous. Potential sources of Cl-VOCs include solvents, pesticides, dry cleaning activities, adhesives, refrigerants, degreasing agents, and poly(vinyl chloride) (PVC) production, among others.^4−6^ For instance, PVC is derived from vinyl chloride; allyl chloride is an intermediate in the production of various chemicals; and 3-chloropropene serves as a solvent and plays a pivotal role in the production of pharmaceuticals, varnishes, adhesives, plastics, and insecticides.^7−9^ In addition, Cl-VOCs are potential Cl atom precursors, which can be transported into the stratosphere and may be involved in the depletion of stratospheric ozone.^10,11^ The presence of Cl-VOCs in the atmosphere can be influenced by wet and dry deposition, photochemical reactions, and interactions with hydroxyl radicals (^•^OH), Cl atoms, and NO_3_ radicals. In addition, if Cl-VOCs contain a double bond, then they may also react with ozone (O_3_) in the atmosphere. There is limited data on the chemical processes involved in the production and removal of these toxic air contaminants, particularly in urban and industrial atmospheres.

Chloroprene (CP; 2-chlorobuta-1,3-diene; CH_2_=C(Cl)–CH=CH_2_) is an important unsaturated chlorinated aliphatic hydrocarbon containing two distinct C=C bonds. It is a major toxic air contaminant. It is released into the gas phase from anthropogenic activities such as neoprene rubber production, with the rubber being used to make wire and cable coatings, oil-resistant rubber materials, and automobile parts.^8,12,13^ Significant amounts of CP can be emitted into the atmosphere during its production, transportation, and usage. Previous studies have highlighted how CP exposure may pose significant risks to human health, notably by elevating the threat of lung and liver cancers.^14,15^ Therefore, it is important to investigate the fate of CP in the environment in order to understand its reactivity and atmospheric transformation mechanisms.

CP at low parts per billion (ppb) levels has been detected in ambient air near industrial sites where it is used.^16^ For example, CP concentrations in the air around Rubbertown in western Louisville, KY, were monitored over a period between 2000 and 2006. Data collected using an EPA procedure (USEPA 2000) showed levels exceeding 1 ppb at a public park and elementary school that were in its vicinity. In another area near the industrial plant, levels of CP peaked at over 10 ppb.^16^

The structure of CP is similar to that of isoprene, with the exception that in the latter the Cl atom is replaced by a methyl group. Plant derived isoprene is well recognized as the dominant non-methane organic compound released into the atmosphere from terrestrial plants. Reports on the atmospheric oxidation of isoprene with OH radical indicate that the reaction proceeds by ^•^OH addition at the terminal C atoms.^17−19^ The rate coefficient for the isoprene + ^•^OH reaction was reported to be 6.0 × 10^–11^ cm^3^ molecule^–1^ s^–1^ at 298 K.^17^ Similar to isoprene, once CP is emitted into the atmosphere, it is expected to react with ^•^OH because ^•^OH is the main daytime oxidant that controls the removal and transformation of pollutants in the atmosphere.^20,21^ The environmental fate of CP is not fully understood, and there is no experimental study available on the oxidation of CP in the gas phase. However, research on the atmospheric chemistry of CP with ^•^OH, ozone, and NO_3_ radicals has been reported.^8,22^ Rate coefficients were estimated using structure–reactivity relationships between rate coefficients and ionization potentials for structural homologues.^8,22^ The rate coefficient for the reaction of CP + ^•^OH was reported to be 6.2 × 10^–11^ cm^3^ molecule^–1^ s^–1^ at 298 K,^8,22^ and a tentative mechanism for the CP + ^•^OH radical reaction has been reported. The mechanism was proposed to involve ^•^OH radical attack preferentially at the C_3_–C_4_ bond, which leads to the formation of formaldehyde, CH_2_=C(Cl)CHO, and ClC(O)CHO as products.^8^

**Figure 1:**
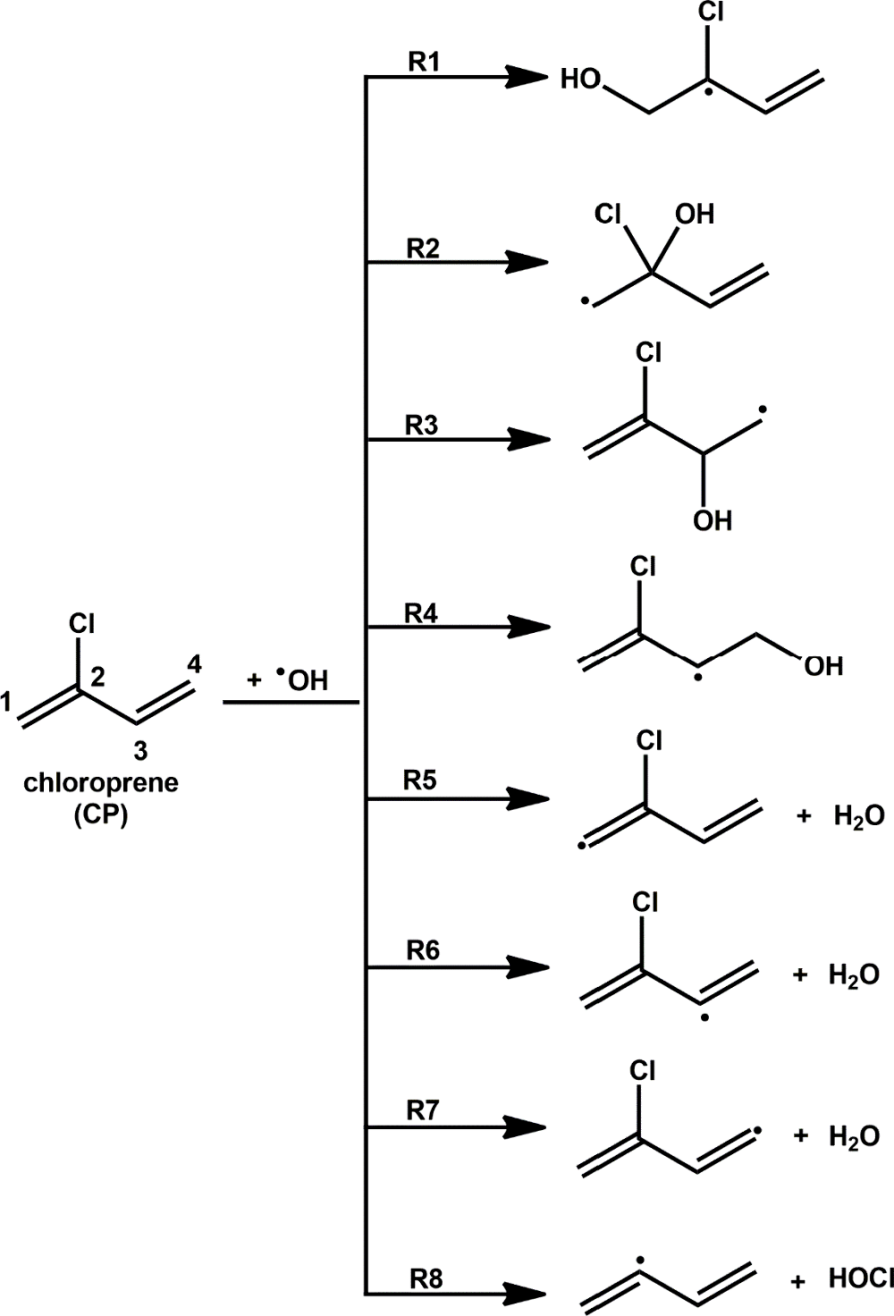
Various possible addition and abstraction paths associated with the reaction of CP + ^•^OH: (i) OH-addition (R1, R2, R3, and R4) paths leading to the formation of the corresponding C-centered CP–OH radicals; (ii) abstraction of an H atom from =CH_2_ and =CH moieties (R5, R6, and R7) and a Cl atom from the =C(Cl) moiety (R8), leading to the formation of the corresponding products (C-centered CP radical + H_2_O/HOCl). The reaction numbering and labeling of the C atoms in CP are also shown.

Due to the presence of unsaturation in the structure of CP, it is anticipated that it would undergo reactions with ^•^OH via addition to sp^2^-hybridized carbon, and hydrogen abstraction paths. The corresponding reaction channels are illustrated in Figure 1, with the C atoms numbered for ease of reference. According to the figure, ^•^OH can attack the carbon atoms of the C_1_–C_2_ and C_3_–C_4_ double bonds, resulting in the formation of carbon-centered CP–OH radical products through reactions R1, R2, R3, and R4. Additionally, ^•^OH can abstract an H atom from the =CH_2_ and =CH moieties of CP, forming their respective carbon-centered CP radicals and H_2_O (see reactions R5, R6, and R7 in Figure 1). Furthermore, ^•^OH can also abstract a Cl atom via reaction R8, resulting in the formation of a C-centered CP radical and HOCl.

In this work, to better understand the atmospheric removal processes, the primary oxidation pathways of CP + ^•^OH were investigated by using *ab initio*/DFT calculations combined with kinetic modeling. All the reaction paths were characterized by optimizing the respective minima and transition states on the potential energy surface (PES). The thermochemistry and accurate kinetics for the various paths were then determined. The rate coefficient calculations for each reaction path were performed using canonical variational transition state theory combined with small curvature tunneling. In addition, we investigated the subsequent reactions of the major product formed in the initial CP + ^•^OH reaction. Finally, we discuss the atmospheric implications. The insights gleaned from this study can contribute to a more complete understanding of the CP + ^•^OH reaction within atmospheric settings. This in turn can enhance the accuracy of models pertaining to regional air quality and lead to a better understanding of the reaction kinetics and dynamics.

## 2 Computational Methods

We used Minnesota hybrid density functional (M06-2X)^23,24^ theory to fully optimize the geometries of all relevant stationary points along the PESs for the addition and abstraction paths of the CP + ^•^OH reaction. The Dunning’s aug-cc-pVTZ basis set was adapted for M06-2X computations (represented as M06-2X/aug-cc-pVTZ).^25^ The M06-2X functional with a variety of basis sets has been used by several researchers who have found it to be appropriate for studying radical + molecule reactions under atmospheric conditions.^26,27^ The geometries optimized at the M06-2X level are provided in Table S1. Using the same level of theory, we performed harmonic vibrational frequency calculations to determine the character of each stationary point. All of the transition states were found to have one imaginary frequency, and all other minima contain all positive vibrational frequencies. Imaginary frequencies of transition states, vibrational frequencies, and rotational constants for all the minima and transition states computed at the M06-2X level are provided in Tables S2, S3, and S4, respectively. In order to gain an understanding of the trajectory of the chemical reaction, we conducted intrinsic reaction coordinate (IRC) calculations to verify the saddle points connected with the respective pre- and postreactive complexes (RCs and PCs) during the course of the reaction.^28,29^ Single point calculations were performed to get more precise energies for all the relevant stationary points on the PESs using the coupled cluster single and double substitution method with a perturbative treatment of triple excitation (CCSD(T))^30^ and the aug-cc-pVTZ basis set on the optimized geometries at the M06-2X/aug-cc-pVTZ computational level. All of the energies were zero point corrected. The zero point energy (ZPE) corrected CCSD(T)/aug-cc-pVTZ//M06-2X/aug-cc-pVTZ (represented as CCSD(T)//M06-2X) level of theory has been successfully used to study ^•^OH reactions with several atmospherically important molecules^31−33^ under tropospheric conditions. The reported rate coefficients using the energies computed at this level agree well with the experimentally measured values. Thermal correction to the enthalpy and thermal correction to the Gibbs free energy were used to calculate enthalpies and Gibbs free energies for all the stationary points on the PES at 298 K. Total electronic energies including ZPE corrections calculated at various levels of theory as well as the thermal correction to the enthalpy and Gibbs free energies computed at the M06-2X level are provided in Table S5. The present *ab initio*/DFT calculations were performed using the Gaussian16 software package.^34^

## 3 Kinetics Calculations

Rate coefficient calculations for reactions between atmospheric molecules and ^•^OH are essential for a complete understanding of atmospheric chemistry, air quality, climate change, and environmental conditions. Therefore, we performed rate coefficient calculations for the reaction of CP + ^•^OH under atmospheric settings. The addition and abstraction paths associated with the CP + ^•^OH reaction were presumed to occur via a two-step complex mechanism shown in eq 1.
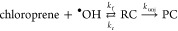
1According to eq 1, fast thermal equilibrium is established between the starting reactants (chloroprene and ^•^OH) and a prereactive complex (RC) in the first step. The formed RC leads to a transition state via unimolecular isomerization to form a postreactive complex (PC) in the second step. This kinetic model was successfully used in previous studies to calculate rate coefficients for atmospheric reactions with an OH radical.^31,35−37^ In eq 1, *k*_f_ and *k*_r_ are the rate coefficients for the forward and reverse reactions in the first step, and *k*_uni_ is the unimolecular rate coefficient for the second step. By applying the pre-equilibrium approximation, the bimolecular rate coefficient (in cm^3^ molecule^–1^ s^–1^) for the reaction given in eq 1 can be written as shown in eq 2.
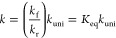
2The symbols *K*_eq_ and *k*_uni_ in eq 2 are the equilibrium constant and the unimolecular rate coefficient, respectively.

The temperature dependent *K*_eq_ can be calculated according to eq 3.
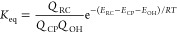
3where the symbols *Q*_RC_, *Q*_CP_, and *Q*_OH_ refer to the product of the individual partition functions of the prereactive complex and starting reactants (CP and ^•^OH), and the symbols *E*_RC_, *E*_CP_, and *E*_OH_ are the ZPE-corrected CCSD(T)//M06-2X energies of the corresponding prereactive complex and the reactants CP and ^•^OH. *R* and *T* are the gas constant and temperature in kelvin (K), respectively.

The unimolecular rate coefficient (*k*_uni_^CVT/SCT^) can be calculated using canonical variational transition state theory^38,39^ (CVTST) combined with a multidimensional small curvature tunneling^40^ (SCT) approximation according to eq 4, as implemented in the Polyrate (2016) program.^41^
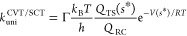
4In eq 4, *Q*_TS_(s*) and *Q*_RC_ are the generalized transition state and prereactive complex partition functions, *V*(s*) is the potential energy at the barrier maximum, the small curvature tunneling parameter is designated as Γ, *k*_B_ is the Boltzmann constant, *h* is Planck’s constant, and *T* is the temperature in K. The rate coefficients were computed with energies obtained at the ZPE-corrected CCSD(T)//M06-2X level, whereas the temperature-dependent equilibrium constants and partition functions were computed using both the CCSD(T)//M06-2X and M06-2X/aug-cc-pVTZ levels.

## 4 Results and Discussion

### 4.1 Conformational Analysis

**Figure 2:**
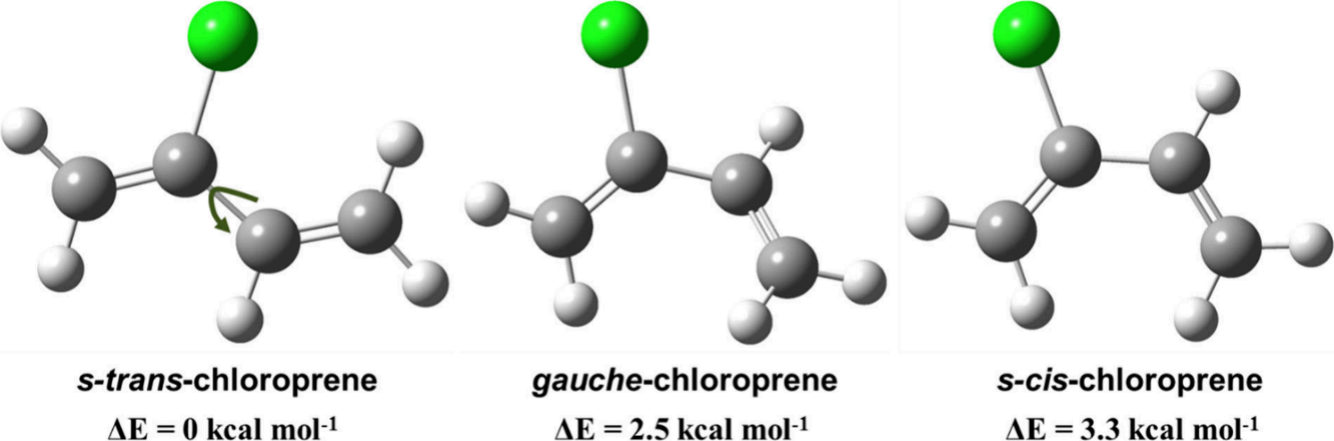
Various possible geometries of CP, optimized at the M06-2X/aug-cc-pVTZ level. The C, H, and Cl atoms are indicated with black, white, and green colors, respectively. The energies of *gauche*-chloroprene and *s-cis*-chloroprene were calculated relative to the energy of the most stable *s-trans*-chloroprene.

Conformational analyses of CP were performed at the M06-2X/aug-cc-pVTZ level through rotation of its central C–C single bond to determine the stable conformers along this degree of freedom. Three stable conformers were found, as depicted in Figure 2. In the *s-trans*-chloroprene conformer, the two double bonds are coplanar and oriented in opposite directions, resulting in a 180° dihedral angle along the C-skeleton. In the gauche conformer, one of the double bonds lies in one plane, while the other is slightly tilted relative to it, resulting in a 38° dihedral angle along the C-skeleton (see Figure 2). In the *s-cis*-chloroprene conformer, both double bonds are in the same plane and point in the same direction, yielding a 0° dihedral angle along the C-skeleton. The relative energies of each conformer calculated at the M06-2X/aug-cc-pVTZ level are listed in Figure 2. The results indicate that the *s-trans*-chloroprene conformer is the most stable, with the *gauche*- and *s-cis*-conformers being 2.5 and 3.3 kcal mol^–1^ higher in energy, respectively, at the same level of theory. Therefore, in this work, the ^•^OH-initiated atmospheric chemistry of CP was studied by considering the structure of the most stable *s-trans-*chloroprene conformer.

**Figure 3:**
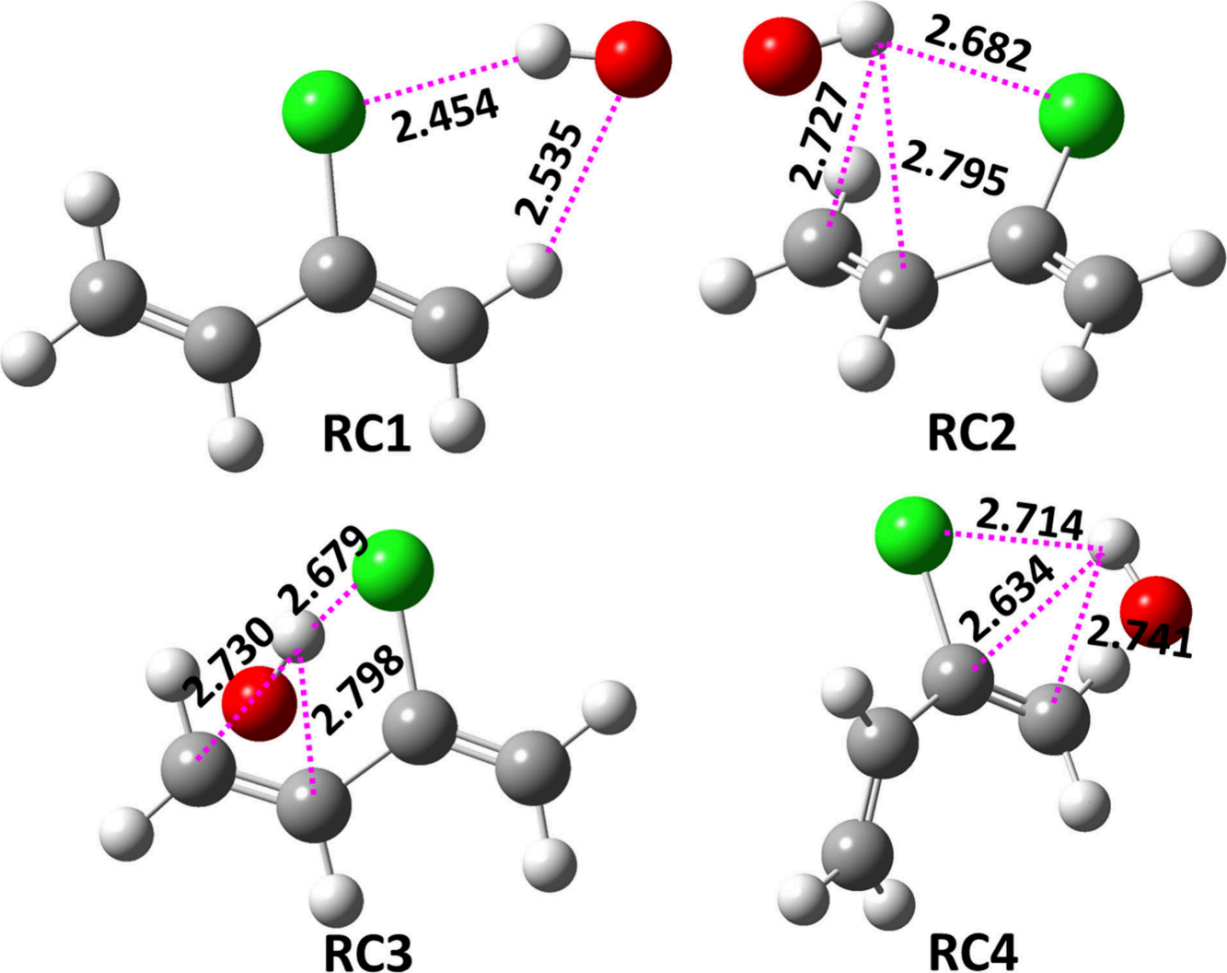
M06-2X/aug-cc-pVTZ optimized structures of the prereactive complexes associated with the CP + ^•^OH reaction. Bond lengths in Å are shown. The H, C, O, and Cl atoms are represented with white, black, red, and green colors, respectively.

Because CP has two distinct double bonds with Cl and H atoms linked to the C atoms, the CP + ^•^OH reaction is expected to proceed via (1) addition and (2) abstraction (see Figure 1). In all the reaction paths, the collision interaction of the initial reactants (^•^OH and CP) primarily proceeds to form four different prereactive complexes (RC1, RC2, RC3, and RC4). The fully optimized geometries of RC1, RC2, RC3, and RC4 at the M06-2X/aug-cc-pVTZ level are shown in Figure 3. The RC1 structure suggests that CP and ^•^OH interact via formation of two hydrogen bonds between the O atom of the ^•^OH and the H atom of the =CH_2_ moiety and a second between the H atom of the ^•^OH and the Cl-atom of CP, with bond lengths of 2.54 and 2.45 Å, respectively. The structures of RC2, RC3, and RC4 suggest that the partial positive charge on the H atom of the polar ^•^OH primarily facilitates its interaction with one of the sites of double bond electron density. The structures of these complexes clearly suggest that the H atom of the ^•^OH approaches the C_3_–C_4_ double bond of CP from above and below the plane in RC2 and RC3, whereas in RC4, the H atom approaches toward the C_1_–C_2_ double bond (see Figure 3). The structures of RC2, RC3, and RC4 also suggest that the ^•^OH is positioned over one of the double bonds, aligning with the CP carbon chain in a nearly parallel plane. All three structures are stabilized by the interaction between the H atom of ^•^OH and the electron density of the respective double bond. In addition, we found hydrogen-bonding interactions between (1) the O atom of the ^•^OH and the =CH_2_ moiety H atoms, and (2) the H atom of the ^•^OH and the Cl atom of CP. These three types of interactions are responsible for the ^•^OH being parallel to the CP carbon chain. The binding energies of RC1, RC2, RC3, and RC4 were found to be −2.4, −2.1, −2.2, and 0.4 kcal mol^–1^ below and above the CP + ^•^OH separated reactants, respectively, obtained at the ZPE-corrected CCSD(T)//M06-2X level. RC1 was found to be slightly more stable than RC2 and RC3. However, structural change in the C_3_–C_4_ double bond in RC4 suggests that the binding energies of RC1, RC2, and RC3 are ∼2.5–2.8 kcal mol^–1^ more stable than the structure of RC4.

### 4.2 Addition Pathways

**Figure 4:**
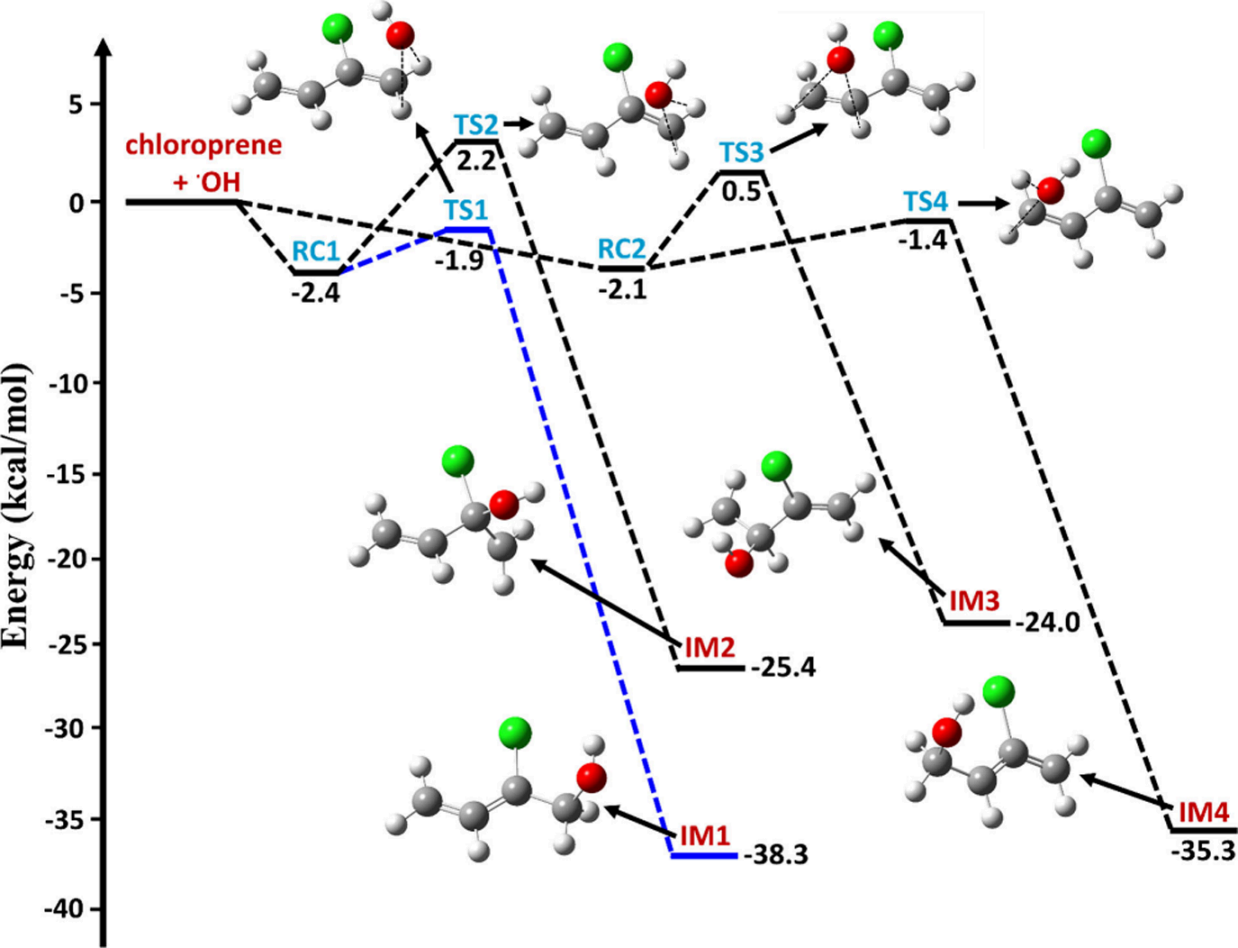
ZPE-corrected CCSD(T)/aug-cc-pVTZ//M06-2X/aug-cc-pVTZ level calculated potential energy profiles for the various addition paths involved in the CP + ^•^OH reaction, leading to the formation of the respective C-centered chloroprene–OH radical products. The symbols RCs, TSs, and IMs represents prereactive complexes, transition states, and C-centered radical products, respectively.

A schematic of the PES profile for the various possible addition pathways for the CP + ^•^OH reaction obtained at the ZPE-corrected CCSD(T)/aug-cc-pVTZ//M06-2X/aug-cc-pVTZ level is shown in Figure 4. According to the figure, ^•^OH attack on the conjugated diene system of CP provides four types of addition paths. The reactions R1 and R2 (see Figures 1 and 4) primarily form barrierless stable RC1, which then proceeds to form TS1 and TS2, with barrier heights of −1.9 and 2.2 kcal mol^–1^ below and above the CP and ^•^OH separated reactants, respectively. The formed TS1 and TS2 further lead to the stable intermediates IM1 (H_2_C=CHC^•^(Cl)CH_2_OH) and IM2 (H_2_C=CHC(OH)(Cl)C^•^H_2_) on the PES at −38.3 and −25.4 kcal mol^–1^ below the starting CP and ^•^OH reactants. Similarly, reaction paths R3 and R4 start from the CP + ^•^OH reactants to form RC2 (see Figures 1 and 4). These reaction paths further lead to the formation of TS3 and TS4 with barrier heights of 0.5 and −1.4 kcal mol^–1^ above and below the energies of the reactants. TS3 and TS4 continue to form IM3 (H_2_^•^CC(OH)(H)C(Cl)=CH_2_) and IM4 (H_2_C(OH)C^•^HC(Cl)=CH_2_) on the PES with energies of −24.0 and −35.3 kcal mol^–1^.

The optimized structures of TS1 and TS2 in Figure 4 indicate that an ^•^OH attack on one of the C atoms of the C_1_–C_2_ double bond of CP results in the formation of a new single bond between the O atom of ^•^OH and the C_1_ atom in TS1 or the C_2_ atom in TS2, followed by slight lengthening of the C_1_–C_2_ bond by 0.02–0.03 Å relative to the bond length of the starting CP reactant. Similarly, the structures of TS3 and TS4 suggest ^•^OH attack on one of the C atoms of the C_3_–C_4_ bond to form a single bond between the O-atom of the ^•^OH and one of the C atoms of the C_3_–C_4_ double bond, which occurs with a simultaneous increase in the bond length of the C_3_–C_4_ bond by 0.02–0.03 Å with respect to the bond length of the starting CP reactant. The bond lengths of the newly formed O–C and C–C bonds are about 2.24 and 1.34 Å in TS1, 2.04 and 1.36 Å in TS2, 2.07 and 1.35 Å in TS3, and 2.20 and 1.34 Å in TS4, respectively. It also suggests that the C=C double bond character is retained in the transition state structures. This is mainly due to the transition states occurring early in the course of all of the CP + ^•^OH addition reactions.

The PES profiles obtained at the CCSD(T)//M06-2X level show that the transition state barriers for the addition of ^•^OH to the terminal C atoms (C_1_ and C_4_) are negative (see Figure 4). The barrier for the addition of ^•^OH to the C_1_ atom via TS1 is about 4.1 and 2.4 kcal mol^–1^ lower than that for the ^•^OH addition to the nonterminal C atoms (i.e., C_2_ and C_3_). The ^•^OH addition to C_4_ via TS4 is about 3.6 and 1.9 kcal mol^–1^ lower than that for the ^•^OH addition to the same nonterminal C atoms (C_2_ and C_3_). This means that ^•^OH preferentially reacts with the terminal carbon atoms of CP rather than the central carbon atoms. Similar behaviors have been observed in the barriers for the reactions of ^•^OH with conjugated double bonded system analogues to CP.^17^ Finally, the results also indicate that the reaction barriers for the addition of ^•^OH to C_1_ and C_4_ are very close. However, the pathway through TS1 is more favorable than the pathway via TS4, as the barrier height for C_1_ addition via TS1 is ∼0.5 kcal mol^–1^ lower than that for C_4_ addition via TS4 (see Figure 4). We also found that the intermediate products IM1 and IM4 formed from TS1 and TS4 are more stable by 13.0–14.3 and 9.9–11.3 kcal mol^–1^ compared to the products IM2 and IM3 formed from TS2 and TS3. The main reason for this high stabilizing effect for IM1 and IM4 is the delocalization of the radical center with the adjacent double bond, a situation that is not possible for IM2 and IM3. These results suggest that ^•^OH addition at either C_2_ or C_3_ of CP is much less important compared to addition to C_1_ and C_4_.

### 4.3 Abstraction Pathways

A schematic representation of PES profiles for H atom abstraction from various C-sites of CP by ^•^OH is shown in Figure S1. The transition state geometries optimized at the M06-2X/aug-cc-pVTZ level and the energies of all of the stationary points on the PES computed at the ZPE-corrected CCSD(T)//M06-2X level are displayed in the figure. A detailed discussion of the PES profiles and energies of all the stationary points for all possible abstraction paths is provided in Section S1 of the Supporting Information. Based on the results (see Figure S1), abstraction of an H atom from the −CH moiety via TS7 with a barrier height of 3.2 kcal mol^–1^ to form IM6 (H_2_C=C(Cl)C^•^=CH_2_) + H_2_O is the most dominant compared to the other possible H and Cl atom abstraction paths. The PES profiles shown in Figure 4 and Figure S1 indicate that the addition of ^•^OH to C_1_ to form CH_2_=CH–C^•^(Cl)–CH_2_OH (IM1) is more dominant compared to all of the other possible addition and abstraction channels associated with the CP + ^•^OH reaction.

We determined the enthalpies and Gibbs free energies (in kcal mol^–1^) of all of the stationary points on the PESs at 298 K for the various possible addition and abstraction paths. The values, which were calculated relative to the starting CP + ^•^OH reactants, are displayed in Table S6. The results show that the addition paths are significantly more spontaneous and thermodynamically favorable than the H-abstraction paths. The enthalpies and Gibbs free energy data also indicate that the addition of ^•^OH via TS1 is energetically favored, with values of −2.5 and 6.0 kcal mol^–1^ below and above the starting reactants, respectively, compared to all other possible addition and abstraction channels.

The calculated enthalpy changes (Δ*H*) for the formation of the addition products IM1, IM2, IM3, and IM4 are −39.3, −26.1, −24.8, and −36.4 kcal mol^–1^, respectively, while the abstraction pathway products (IM5 + H_2_O, IM6 + H_2_O, IM7 + H_2_O, and IM8 + HOCl) have Δ*H* values of −3.7, −6.8, −3.7, and 32.5 kcal mol^–1^, respectively. These enthalpy values indicate that the formation of products from the addition channels is highly exothermic compared to the abstraction channels. Moreover, the calculated Gibbs free energy changes (Δ*G*) for the formation of IM1, IM2, IM3, and IM4 are −29.7, −17.1, −15.4, and −26.5 kcal mol^–1^, respectively, whereas the abstraction pathway products (IM5 + H_2_O, IM6 + H_2_O, IM7 + H_2_O, and IM8 + HOCl) have Δ*G* values of −5.3, 0.0, −5.3, and 29.2 kcal mol^–1^, respectively. The highly negative Gibbs free energy values further confirm that the addition reactions are more spontaneous than the abstraction pathways.

### 4.4 Kinetics for the Reaction of CP with ^•^OH

The reaction between CP and ^•^OH is intricate, featuring multiple pathways and various transition states that yield different products. To streamline the analysis, we assumed that once a pathway is initiated, it progresses independently without interacting or crossing over with other pathways. We first calculated the temperature-dependent equilibrium constant for the first step and the unimolecular rate coefficients for the second step (see eq 1) involving all possible addition and abstraction channels associated with the CP + ^•^OH reaction in the temperature range of 200 to 400 K. The obtained values are given in Tables S7 and S8. The bimolecular rate coefficients for each pathway were calculated by using the corresponding values of *K*_eq_ and *k*_uni_ at the respective temperature using eq 2. The obtained bimolecular rate coefficient values in the temperature range of 200 to 400 K are displayed in Table 1. The results suggest that the values of the rate coefficients decrease with increasing temperature (i.e., a negative temperature dependence trend) for the reaction paths via TS1 and TS4 in the current studied temperature range. In contrast, the rate coefficients for the other possible addition channels that proceed via TS2 and TS3 were found to increase within the studied temperature range (i.e., positive temperature dependence trend). The negative and positive temperature dependence trends occur because the addition reactions through TS1 and TS4 have a substantial negative energy barrier, whereas the addition pathways proceeding through TS2 and TS3 possess a positive barrier. In addition, a further contributor to the negative temperature dependence of the rate coefficients for TS1 and TS4 is the presence of barrierless prereactive complexes in the first step of the CP + ^•^OH reaction, which results in negative transition state barriers. Such reaction paths exhibit this trend; several studies have previously shown that OH-addition reactions to alkenes, substituted alkenes, and aromatic hydrocarbons involve prereactive complexes in their reaction mechanisms, and the reported rate coefficients exhibit a negative temperature dependence.^42−44^ The data from the table indicate that the rate coefficient for the addition of ^•^OH to C_1_ of CP via TS1 to form IM1 is 3–5, 2–3, and 1 order of magnitude larger than that for the ^•^OH addition at C_2_, C_3_, and C_4_ of CP via TS2, TS3, and TS4 to form IM2, IM3, and IM4, respectively, in the present studied temperature range. For example, the bimolecular rate coefficient for TS1 was found to be 1.3 × 10^–10^ cm^3^ molecule^–1^ s^–1^ at 298 K, which is almost 4, 4, and 2 orders of magnitude larger compared to the reaction paths proceeding through TS2, TS3, and TS4 at the same temperature.

**Table 1:** Bimolecular Rate Coefficients (cm^3^ molecule^–1^ s^–1^) for the Various Possible Addition and Abstraction Paths and Overall Rate Coefficients for the CP + ^•^OH Reaction in the Temperatures between 200 and 400 K

*T* (K)	TS1	TS2	TS3	TS4	TS5	TS6	TS7	TS8	TS9	TS10	*k*_overall_
200	3.85 × 10^–10^	3.85 × 10^–15^	5.29 × 10^–14^	1.18 × 10^–11^	4.36 × 10^–17^	3.94 × 10^–30^	1.89 × 10^–12^	1.77 × 10^–18^	6.46 × 10^–19^	2.38 × 10^–50^	3.99 × 10^–10^
210	3.23 × 10^–10^	4.88 × 10^–15^	5.61 × 10^–14^	1.01 × 10^–11^	6.54 × 10^–17^	2.93 × 10^–30^	1.52 × 10^–12^	2.83 × 10^–18^	1.04 × 10^–18^	1.54 × 10^–48^	3.35 × 10^–10^
220	2.79 × 10^–10^	6.12 × 10^–15^	5.94 × 10^–14^	8.81 × 10^–12^	9.60 × 10^–17^	2.24 × 10^–30^	1.25 × 10^–12^	4.39 × 10^–18^	1.63 × 10^–18^	6.85 × 10^–47^	2.89 × 10^–10^
230	2.43 × 10^–10^	7.54 × 10^–15^	6.26 × 10^–14^	7.80 × 10^–12^	1.38 × 10^–16^	1.76 × 10^–30^	1.05 × 10^–12^	6.63 × 10^–18^	2.49 × 10^–18^	2.21 × 10^–45^	2.52 × 10^–10^
240	2.17 × 10^–10^	9.15 × 10^–15^	6.61 × 10^–14^	6.99 × 10^–12^	1.95 × 10^–16^	1.40 × 10^–30^	9.00 × 10^–13^	9.75 × 10^–18^	3.72 × 10^–18^	5.36 × 10^–44^	2.25 × 10^–10^
250	1.95 × 10^–10^	1.10 × 10^–14^	6.96 × 10^–14^	6.35 × 10^–12^	2.69 × 10^–16^	1.15 × 10^–30^	7.84 × 10^–13^	1.40 × 10^–17^	5.45 × 10^–18^	1.01 × 10^–42^	2.02 × 10^–10^
260	1.77 × 10^–10^	1.32 × 10^–14^	7.32 × 10^–14^	5.81 × 10^–12^	3.66 × 10^–16^	9.56 × 10^–31^	6.94 × 10^–13^	1.98 × 10^–17^	7.81 × 10^–18^	1.53 × 10^–41^	1.83 × 10^–10^
270	1.62 × 10^–10^	1.55 × 10^–14^	7.68 × 10^–14^	5.39 × 10^–12^	4.90 × 10^–16^	8.05 × 10^–31^	6.21 × 10^–13^	2.73 × 10^–17^	1.10 × 10^–17^	1.90 × 10^–40^	1.68 × 10^–10^
280	1.50 × 10^–10^	1.81 × 10^–14^	8.05 × 10^–14^	5.02 × 10^–12^	6.45 × 10^–16^	6.91 × 10^–31^	5.63 × 10^–13^	3.71 × 10^–17^	1.51 × 10^–17^	1.98 × 10^–39^	1.56 × 10^–10^
290	1.41 × 10^–10^	2.11 × 10^–14^	8.43 × 10^–14^	4.72 × 10^–12^	8.39 × 10^–16^	6.03 × 10^–31^	5.15 × 10^–13^	4.95 × 10^–17^	2.06 × 10^–17^	1.76 × 10^–38^	1.46 × 10^–10^
298.15	1.33 × 10^–10^	2.36 × 10^–14^	8.74 × 10^–14^	4.51 × 10^–12^	1.03 × 10^–16^	5.42 × 10^–31^	4.83 × 10^–13^	6.20 × 10^–17^	2.62 × 10^–17^	9.43 × 10^–38^	1.38 × 10^–10^
300	1.32 × 10^–10^	2.43 × 10^–14^	8.82 × 10^–14^	4.47 × 10^–12^	1.08 × 10^–16^	5.28 × 10^–31^	4.76 × 10^–13^	6.51 × 10^–17^	2.76 × 10^–17^	1.36 × 10^–37^	1.37 × 10^–10^
400	8.71 × 10^–11^	7.39 × 10^–14^	1.27 × 10^–13^	3.20 × 10^–12^	7.84 × 10^–15^	5.31 × 10^–31^	3.11 × 10^–13^	5.57 × 10^–16^	2.72 × 10^–16^	4.18 × 10^–31^	9.08 × 10^–11^

The bimolecular rate coefficient values for all possible H-abstraction pathways associated with the CP + OH radical reaction, calculated in the temperature range of 200 to 400 K, are displayed in Table 1. The data in Table 1 suggest that the rate coefficients for the reaction pathways proceeding through TS5, TS8, TS9, and TS10 increase with increasing temperature, demonstrating a positive temperature dependence. Conversely, the abstraction channel that proceeds via TS7 exhibits a decrease in the rate coefficients with increasing temperature, reflecting a negative temperature dependence within the studied temperature range. The difference in the rate coefficient trend for these reaction paths is mainly because H-abstraction proceeding via TS5, TS8, TS9, and TS10 exhibits significantly larger barriers than the H-abstraction that proceeds via TS7, which has a significantly lower barrier. Surprisingly, the rate coefficients for H-abstraction via TS6 were found to exhibit a negative temperature dependence in the studied temperature range, even though this reaction path has a significantly larger barrier compared to those of other possible H-abstraction paths. Further, we have calculated the rate coefficients for this reaction beyond 400 K to see the trend in the rate coefficients and found that they started to increase with increasing temperature. The rate coefficients for the abstraction of an H and Cl atom from the =CH_2_ and =C(Cl) moieties of CP via TS6 and TS10, respectively, were found to be insignificant within the present investigated temperature range (see Table 1). This is mainly due to the barrier heights for these two paths being significantly larger. Hence, they are not feasible under atmospheric conditions. The data also indicate that the major H-abstraction path occurs via TS7, with a rate coefficient at 298 K of 4.8 × 10^–13^ cm^3^ molecule^–1^ s^–1^, which is 3, 4, and 4 orders of magnitude larger compared to the values for TS5, TS8, and TS9, respectively, at the same temperature. The rate coefficient for the major addition path via TS1 was found to be 2 orders of magnitude larger compared to the values for the major H-abstraction path via TS7 in the present studied temperature range. The energy and rate coefficient results indicate that ^•^OH addition to the C_1_ atom of CP to form OHCH_2_–C^•^(Cl)–CH=CH_2_ (IM1) is a dominant channel compared with the others under atmospherically relevant conditions.

The overall rate coefficient, indicating the rate at which ^•^OH disappears, can be established by aggregating the rate coefficients calculated for various reaction channels at the corresponding temperatures. The obtained temperature-dependent overall rate coefficients in the temperature range of 200–400 K are given in Table 1. The data in the table suggest that the overall rate coefficients decrease with increasing temperature. For example, the overall rate coefficients for the CP + ^•^OH reaction at 200 and 298 K were found to be 4.0 × 10^–10^ and 1.4 × 10^–10^ cm^3^ molecule^–1^ s^–1^, respectively. The reported rate coefficient^8,22^ for the CP + ^•^OH reaction at 298 K was 6.2 × 10^–11^ cm^3^ molecule^–1^ s^–1^, which is ∼2 times smaller compared to the present calculated value at the same temperature. We note that the present calculated rate coefficient for the CP + ^•^OH reaction at 298 K is almost ∼2 times larger than that of the analogous isoprene + ^•^OH reaction which has a rate coefficient of 6.0 × 10^–11^ cm^3^ molecule^–1^ s^–1^ at the same temperature.^17^

### 4.5 Subsequent Reactions of HOCH_2_C^•^(Cl)CH=CH_2_

The calculated energies and kinetics results suggest that the reaction of CP + ^•^OH proceeds through an addition path that forms IM1 as a primary product, releasing 38.3 kcal mol^–1^ of energy (see Figure 4). Accordingly, it is important to determine whether self-isomerization of chemically activated IM1 is feasible under atmospheric conditions. Figure S2 illustrates the PES profile for the self-isomerization process of IM1 calculated at the ZPE-corrected CCSD(T)//M06-2X level. The results show that the barrier for this reaction via TS11 is ∼53.5 kcal mol^–1^ relative to the energy of the IM1. The formed TS11 further leads to the formation of IM9 (cyc-C_3_H_4_Cl–CH_2_OH radical). However, the barrier height for TS11 indicates that this reaction would be negligible under tropospheric conditions due to its significantly high barrier (Figure S2). Thus, the formed IM1 radical from the CP + ^•^OH reaction can undergo (1) direct hydrogen abstraction and (2) addition of ^3^O_2_ under tropospheric conditions. We confirmed that the direct hydrogen abstraction reactions are relatively minor channels based on the results of previous studies involving analogous reactions. The estimated rate coefficients for addition of O_2_ to IM1 and direct hydrogen abstraction are 3 × 10^–12^ and 8 × 10^–15^ cm^3^ molecule^–1^ s^–1^, respectively. These values are derived from the addition of ^3^O_2_ to the C-centered isoprene–OH radical and direct H-abstraction from the C-centered isoprene–OH radical by ^3^O_2_.^45^ Therefore, once formed, IM1 can rapidly react with ^3^O_2_ to form the corresponding CP–OH-derived peroxy (RO_2_) radicals.

**Figure 5:**
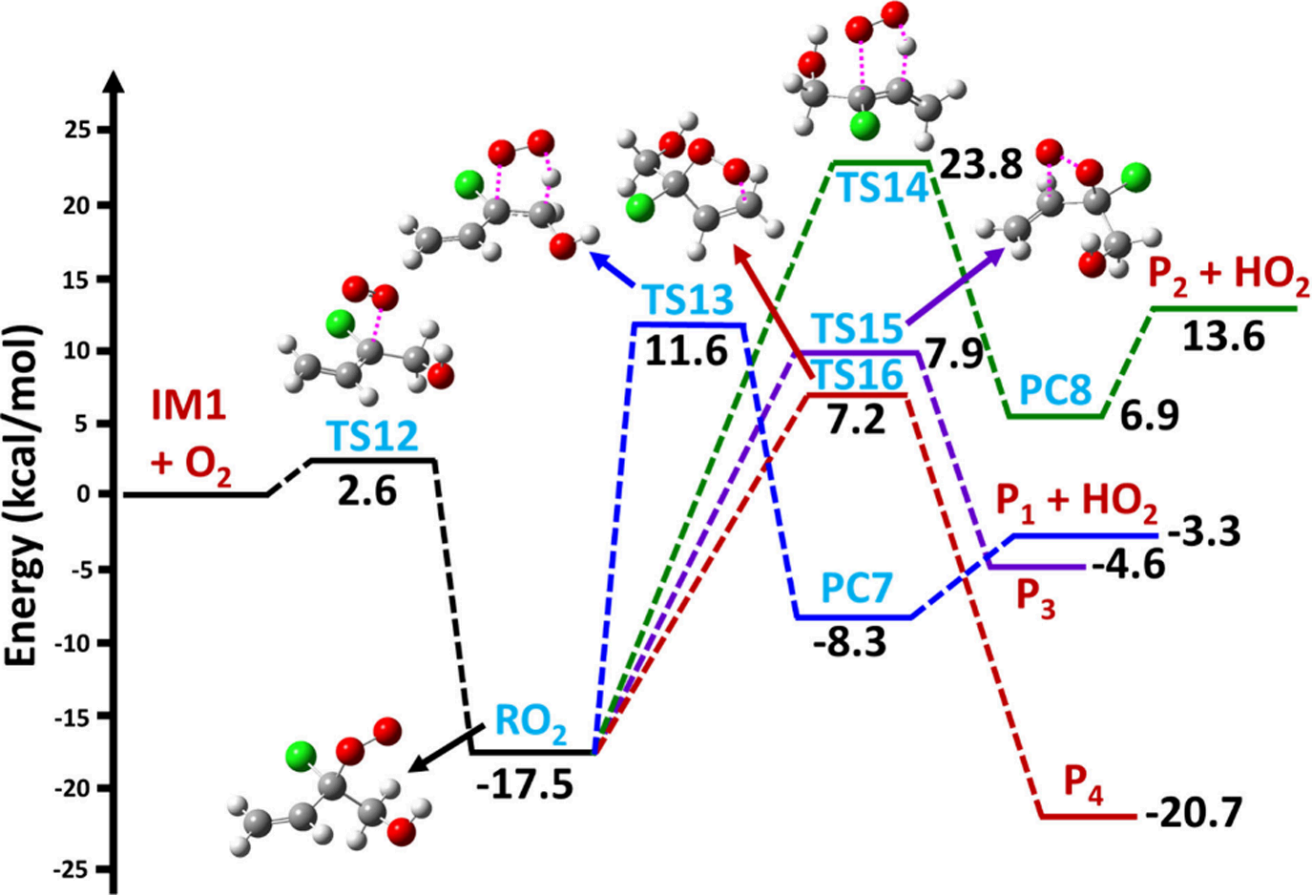
ZPE-corrected CCSD(T)/aug-cc-pVTZ//M06-2X/aug-cc-pVTZ level calculated PES profiles for the HO_2_ elimination and cyclization reactions of the RO_2_ radical adduct formed from the IM1 + ^3^O_2_ reaction leading to the formation of various products. The symbols IM1 and RO_2_ represent the CH_2_=CHC^•^(Cl)CH_2_OH and CH_2_=CHC(OO^•^)(Cl)CH_2_OH adducts, respectively; TS12, TS13, TS14, TS15, and TS16 represent transition states; P_1_ (CH_2_=CHC(Cl)=CHOH), P_2_ (HOCH_2_C(Cl)=C=CH_2_), P_3_ (^•^CH_2_–cyc-C_2_O_2_HCl–CH_2_OH), and P_4_ (HOCH_2_−(cyc-C_3_O_2_ClH_3_)^•^) represent products.

**Figure 6:**
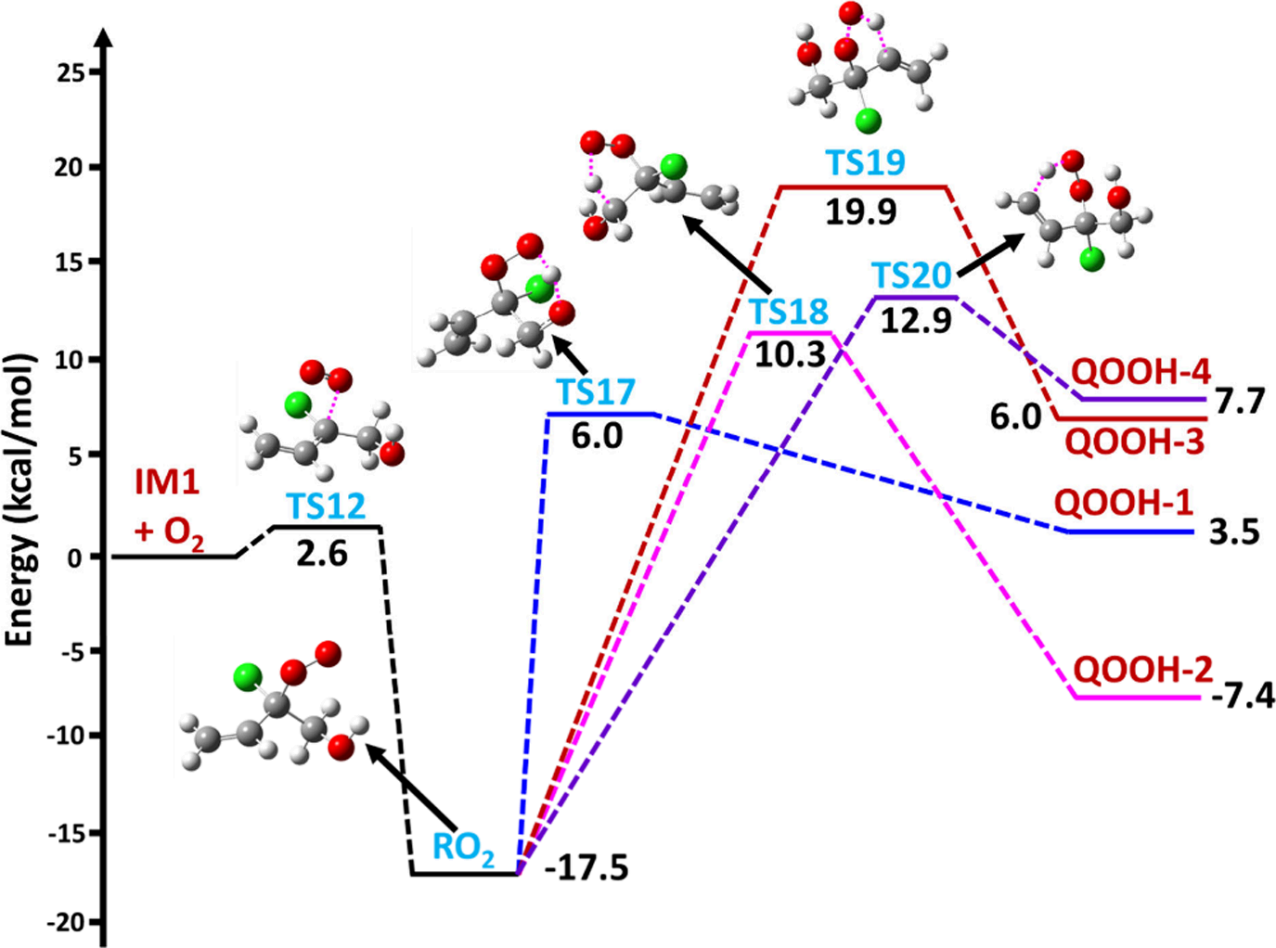
ZPE-corrected CCSD(T)/aug-cc-pVTZ//M06-2X/aug-cc-pVTZ level calculated PES profile for the hydrogen transfer reactions of the RO_2_ radical adduct formed in the reaction IM1 + O_2_ reaction. The symbols IM1 and RO_2_ represent the CH_2_=CHC^•^(Cl)CH_2_OH and CH_2_=CHC(OO^•^)(Cl)CH_2_OH adducts, respectively; TS17, TS18, TS19, and TS20 represent transition states; QOOH-1 (^•^OCH_2_C(OOH)ClCH=CH_2_), QOOH-2 (HOC^•^HC(OOH)(Cl)CH=CH_2_), QOOH-3 (HOCH_2_C(OOH)(Cl)C^•^=CH_2_), and QOOH-4 (HOCH_2_C(OOH)(Cl)CH=C^•^H) represent products.

The PES profile for the IM1 + ^3^O_2_ reaction leading to the formation of the respective RO_2_ radical adduct is shown in Figures 5 and 6. The energies computed for various stationary points on the PES at the ZPE-corrected CCSD(T)//M06-2X levels are also shown in the figures. It can be noted from the results that the IM1 + ^3^O_2_ reaction primarily leads to the formation of a transition state (TS12) with a barrier height of 2.6 kcal mol^–1^ above that of the separated IM1 and O_2_ reactants. The TS12 structure indicates that the addition of the O_2_ occurs at the C-site of HOCH_2_C^•^(Cl)CH=CH_2_ (see Figures 5 and 6). The formed TS12 further leads to a stable RO_2_ radical adduct with a binding energy of −17.5 kcal mol^–1^ that is below that of the IM1 and ^3^O_2_ reactants. This binding energy agrees reasonably well with the value for the analogous reaction reported for isoprene to yield the isoprene–OH–O_2_ adduct (∼18 kcal mol^–1^).^46^ It is also important to note that the addition of ^3^O_2_ to allyl-like radicals (IM1) to form RO_2_ radical adducts is notably less exothermic compared to typical alkyl + ^3^O_2_ reactions to form the corresponding RO_2_ radical adducts (30–35 kcal mol^–1^) due to the absence of allyl resonance stabilization.^47^

### 4.6 Unimolecular Reactions of the RO_2_ Radical Adduct

According to various studies,^48,49^ typical RO_2_ radicals can undergo two possible types of competitive reactions, namely unimolecular and bimolecular reactions of RO_2_ with NO and HO_2_ radicals. The unimolecular reactions of the RO_2_ radical adduct include such transformations as HO_2_ elimination, cyclization, and H atom transfer, which are shown in Figures S3 and S4, respectively. A schematic representation of the PES profiles for these reaction paths is also shown in Figures 5 and 6. In these figures, the various possible transition state structures for the HO_2_ elimination, cyclization, and H atom transfers and their corresponding stationary point energies calculated at the ZPE-corrected CCSD(T)//M06-2X level are displayed. Two types of unimolecular HO_2_ elimination, two types of cyclization and four types of intramolecular hydrogen atom transfer reactions were identified involving the RO_2_ radical adduct. The results for the unimolecular HO_2_ elimination reactions suggest that H atom transfer from the −CH_2_ and =CH moieties of the RO_2_ radical adduct to the terminal O atom, followed by C–O single bond scission via TS13 and TS14 (with a barrier height of 11.6 and 23.8 kcal mol^–1^, respectively), relative to the IM1 + ^3^O_2_ separated reactants (see Figure 5) occurs. The formed TS13 and TS14 proceed to form PC7 and PC8, which then leads to the formation of P_1_ (H_2_C=CHC(Cl)=CHOH) + HO_2_ and P_2_ (H_2_C=C=C(Cl)CH_2_OH) + HO_2_ products, respectively. Based on the energetics, formation of P_1_ + HO_2_ via TS13 has an ∼12.2 kcal mol^–1^ lower barrier compared to the formation of P_2_ + HO_2_ via TS14. The cyclization reactions proceed via TS15 and TS16 with barrier heights of 7.9 and 7.2 kcal mol^–1^ to form the respective C-centered four-membered (P_3_, ^•^CH_2_–cyc-C_2_O_2_HCl–CH_2_OH) and five-membered (P_4_; HOCH_2_−(cyc-C_3_O_2_ClH_3_)^•^) cyclic radical products, respectively. The structures of TS15 and TS16 on the PES indicate that the terminal O atom of the RO_2_ radical attacks the nonterminal C_3_ and terminal C_4_ atoms, leading to formation of a new single bond between the O atom and the respective C atoms (i.e., P_3_ and P_4_), respectively. Based on the energetics, formation of the C-centered five-membered radical product (P_4_) is a major path that is slightly more favored compared to the formation of the C-centered four-membered radical product (see Figure 5).

The PES profiles for the intramolecular H atom transfer reactions indicate that the H atoms from the −OH, −CH_2_, =CH, and =CH_2_ moieties are shifted to the terminal O atom of the R–OO moiety. There are two different 1,5 H atom transfer paths, where an H atom shifts from the −OH and =CH_2_ moieties of RO_2_ to the terminal −OO group. The remaining two different 1,4 H atom transfers involve shifts from the −CH_2_ and =CH moieties of RO_2_ to the terminal −OO group (see Figure 6 and Figure S4). All four of these H atom transfer reactions proceeded to the formation of O- and C-centered QOOH radicals (see Figure S4). The results as shown in the figures suggest that the 1,5 H atom transfer via TS17 to form the O-centered QOOH radical is the most favorable pathway since the other H atom transfer channels have higher barriers. The energetics of all other possible unimolecular reactions of the RO_2_ radical adduct indicate that the 1,5-H atom transfer to form the O-centered QOOH radical is the major reaction compared to the other possible H atom transfers or the cyclization and direct HO_2_ elimination pathways.

### 4.7 Kinetics for the Reaction of IM1 + ^3^O_2_

The competition between bimolecular (RO_2_ + NO/HO_2_) and RO_2_ unimolecular reactions was mainly influenced by the kinetics of the unimolecular reactions along with the concentrations of NO and HO_2_ radicals. Consequently, understanding the unimolecular reaction mechanisms and kinetics is crucial for determining the atmospheric behavior of CP–OH-derived RO_2_ radical adducts. Therefore, we performed kinetics analysis of the IM1 + ^3^O_2_ reaction using the Master Equation Solver for Multi-Energy Well Reactions (MESMER) kinetics program.^50^ Several studies have effectively utilized it to examine the kinetics of O_2_ reactions with different compounds.^48,49,51,52^ Rate coefficients for the present studied unimolecular reactions featuring a well-defined transition state were determined using the Rice–Ramsperger–Kassel–Marcus (RRKM) theory. Input parameters such as rotational constants, vibrational frequencies, and energies for all of the stationary points on the PESs (see Figures 5 and 6) for the MESMER modeling were obtained from the M06-2X/aug-cc-pVTZ and the ZPE-corrected CCSD(T)//M06-2X level of calculations. Rate coefficients for the barrierless reactions involved in direct HO_2_ elimination (from products to PC) were obtained via the inverse Laplace transformation (ILT) method. In the ILT approach, the temperature-independent capture rate coefficient of 1.0 × 10^–11^ cm^3^ molecule^–1^ s^–1^ was used. Nitrogen (N_2_) served as the buffer gas in the Mesmer simulations. The single-exponential down model with an average transfer energy of Δ*E*_d_ = 200 cm^–1^ was used to simulate the collision energy transfer between active intermediates and N_2_. Lennard-Jones parameters for intermediates were based on those of the nearest sized alkane (ε = 306.5 K, σ = 4.4 Å).^53^ Tunneling corrections were incorporated in the rate coefficient calculations for reactions involving hydrogen atom transfer, cyclization, and direct HO_2_ elimination, using a one-dimensional unsymmetrical Eckart barrier.^54^

Rate coefficients were calculated using the PES profiles for the various possible unimolecular reactions involving the IM1 + ^3^O_2_ reaction (see Figures 5 and 6). The obtained pseudo-first-order rate coefficient at 298 K for the direct HO_2_ elimination pathways (IM1 + O_2_ → RO_2_ → P_1_ + HO_2_ and IM1 + O_2_ → RO_2_ → P_2_ + HO_2_) were found to be 3.2 × 10^–8^ and 4.4 × 10^–16^ s^–1^, respectively. Based on the rates, the formation of P_1_ + HO_2_ was found to be ∼7.3 × 10^7^ times more preferred compared to the formation of the P_2_ + HO_2_ products from the same reactants. The calculated pseudo-first-order rate coefficients at 298 K for the cyclization reactions (IM1 + O_2_ → RO_2_ → P_3_ and IM1 + O_2_ → RO_2_ → P_4_) are found to be 3.8 × 10^–7^ and 5.1 × 10^–7^ s^–1^, respectively. This suggests that formation of the five-membered ring radical (P_4_) product is slightly more preferred (∼1.3 times) compared to the four-membered ring radical (P_3_) product. We also calculated the pseudo-first-order rate coefficient for the various possible hydrogen shift reactions (IM1 + O_2_ → RO_2_ → QOOH-1; IM1 + O_2_ → RO_2_ → QOOH-2; IM1 + O_2_ → RO_2_ → QOOH-3; and IM1 + O_2_ → RO_2_ → QOOH-4), and the corresponding values at 298 K were found to be 7.6 × 10^–9^, 3.8 × 10^–7^, 4.6 × 10^–12^, and 5.3 × 10^–11^ s^–1^, respectively. The results indicate that formation of the QOOH-2 radical is preferred by ∼2–5 orders of magnitude when compared to the formation of other possible QOOH radical products. These results also indicate that the cyclization and hydrogen atom transfer reactions are more dominant by ∼1–9 orders of magnitude compared to the direct HO_2_ elimination reactions.

### 4.8 Subsequent Transformations of RO_2_ with NO and HO_2_

Unimolecular reactions of RO_2_ radical adducts formed from IM1 + ^3^O_2_-type reactions often occur in the midst of competition involving NO and HO_2_ radicals. Therefore, we also considered bimolecular reactions of the CP-derived RO_2_ radical adduct with the NO and HO_2_ radical. The corresponding RO_2_ + NO and RO_2_ + HO_2_ reactions which lead to formation of the corresponding alkoxy radical and hydroperoxide are shown in Figure S5. Typical RO_2_ + NO and RO_2_ + HO_2_ reaction rate coefficients are reported to be 9.0 × 10^–12^ and 1.7 × 10^–11^ cm^3^ molecule^–1^ s^–1^, respectively.^48,55,56^ Using these bimolecular rate coefficients along with the concentrations of NO and HO_2_ that are typical in remote and urban atmospheres in the afternoon (i.e., 100 and 50 ppt, respectively),^48,57^ we determined the pseudo-first-order rate coefficients for the reactions RO_2_ + NO and RO_2_ + HO_2_ to be 0.023 and 0.017 s^–1^, respectively. These values suggest the rates of RO_2_ + NO and RO_2_ + HO_2_ bimolecular reactions are more dominant by ∼5 orders of magnitude compared to unimolecular reactions such as cyclization and hydrogen atom shifts.

Interestingly, low NO levels of ∼50 ppt have been found in indoor environments such as museums, classrooms, and a university gym, provided there is no significant external source of NO, and the O_3_ concentration exceeds roughly 10 ppb.^58−61^ Additionally, under typical indoor conditions, HO_2_ radical concentrations of 4 × 10^7^ molecules cm^–3^ have been reported.^62^ Thus, pseudo-first-order rate coefficients for RO_2_ + NO and RO_2_ + HO_2_ reactions to form the corresponding alkoxy radicals and hydroperoxides under these atmospheric conditions were also calculated, and the corresponding values were found to be 1.1 × 10^–2^ and 6.8 × 10^–4^ s^–1^. This suggests that even in typical indoor environments, bimolecular CP–OH-derived RO_2_ + NO and RO_2_ + HO_2_ radical reactions are more dominant when compared to CP–OH-derived RO_2_ unimolecular decomposition reactions.

## 5 Conclusion

We used an ^•^OH concentration of ∼1.0 × 10^6^ radicals cm^–3^ and the overall rate coefficients for the CP + ^•^OH reaction calculated in the present study in the temperatures between 200 and 400 K (see Table 1) to calculate the atmospheric lifetime of CP with respect to its reaction with ^•^OH. The atmospheric lifetime of CP due to loss by reactions with ^•^OH is estimated to be ∼1–2 days in the present studied temperatures between 200 and 400 K. The life span of CP is an estimate only, as its degradation is significantly influenced by the specific timing and location of its release, as well as the fluctuating levels of ^•^OH in the environment.

The CP + O_3_ and CP + NO_3_ reaction rate coefficients at 298 K were previously reported to be 1.4 × 10^–16^ and 3.6 × 10^–13^ cm^3^ molecule^–1^ s^–1^, respectively.^8^ We estimated the atmospheric lifetime of CP with respect to the reactions with O_3_ and NO_3_ at 298 K using the reported rate coefficients. We also used the average concentration of [O_3_] = 1.0 × 10^12^ molecules cm^–3^ and [NO_3_] = 2.0 × 10^8^ molecules cm^–3^.^63,64^ We found that the atmospheric lifetimes of CP with respect to those of O_3_ and NO_3_ were 2.0 and 4.0 days, respectively. This indicates that the most important tropospheric sink for CP is its reactions with OH, O_3_, and NO_3_ radicals. The lifetime of CP with respect to its reactions with ^•^OH, O_3_, and the NO_3_ radical was found to be very short, which suggests that it has limited global warming potential (GWP).

**Figure 7:**
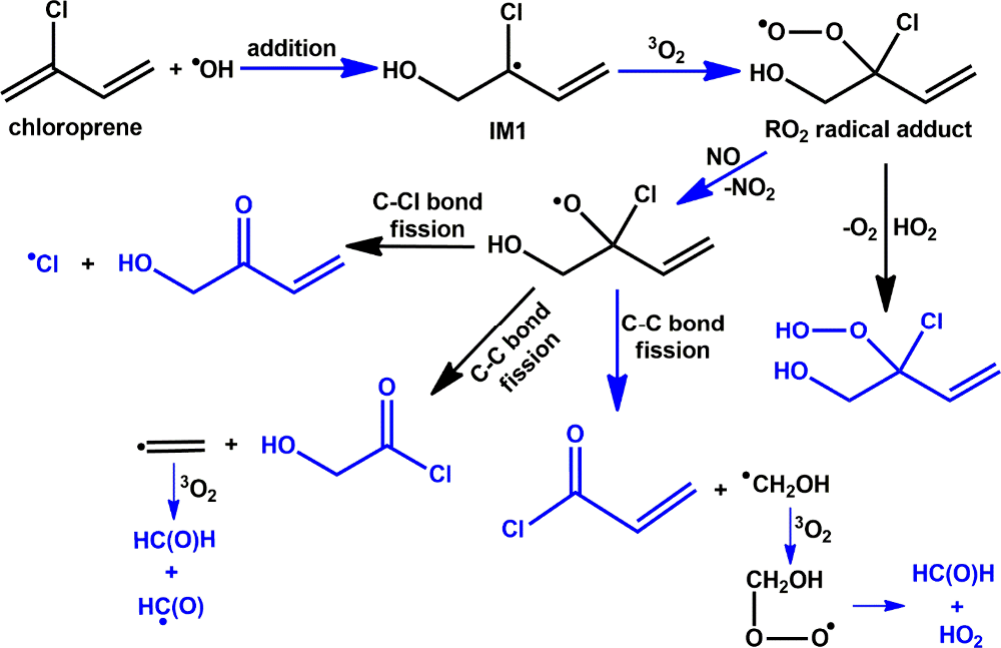
Primary reaction pathways for the atmospheric transformation of CP in the presence of ^•^OH, followed by subsequent reactions of HOCH_2_C^•^(Cl)CH=CH_2_ with ^3^O_2_, HO_2_ radical, and NO. This process leads to the formation of various compounds, including HOCH_2_C(OOH)(Cl)CH=CH_2_, HC(O)H, HO_2_ radical, ClC(O)CH=CH_2_, HOCH_2_C(O)Cl, HC(O) radical, Cl atom, and HOCH_2_C(O)CH=CH_2_. The final products formed under tropospheric conditions are highlighted in blue.

Based on the aforementioned results, we have proposed a mechanism for the CP + ^•^OH reaction and its corresponding RO_2_ radical adduct under tropospheric conditions. This is presented in Figure 7. It suggests that ^•^OH attack occurs at the four sp^2^-hybridized carbons of the diene, with a preference for the terminal C_1_ carbon atom yielding a C-centered radical (IM1; (HOCH_2_C^•^(Cl)CH=CH_2_)) at the carbon harboring the chlorine atom. The formed IM1 radical can combine with ^3^O_2_ leading to a peroxy radical adduct (RO_2_) under atmospheric conditions (see Figure 7). Of the subsequent reaction paths available to the adduct, H atom transfer, cyclization, and direct HO_2_ elimination were found to be minor channels in both outdoor and indoor atmospheric environments. Hence, RO_2_ radicals proceed to react with NO and HO_2_ radicals under high NO and HO_2_ radical atmospheric conditions. As shown in Figure 7, the engagement of RO_2_ radical adducts with HO_2_ radicals forms the corresponding alkyl hydroperoxides + HO_2_ radical products. On the other hand, the RO_2_ radical adduct can react with NO, leading to formation of the corresponding alkoxy radical + NO_2_. The formed alkoxy radical (H_2_C=CHC(Cl)(O^•^)CH_2_OH; RO^•^) further undergoes two C–C and one C–Cl bond scission as suggested in Figure 7. The PES profiles for the unimolecular decomposition of RO^•^ calculated at the ZPE-corrected CCSD(T)//M06-2X level is shown in Figure S6. The results from the figure suggest that RO^•^ undergoes C–CH_2_OH bond scission via TS21 with a barrier height of ∼2.9 kcal mol^–1^, which then leads to the formation of PC9, followed by the formation of CH_2_=CHC(O)Cl + ^•^CH_2_OH as products. It can also undergo C–CH bond scission via TS22 with a barrier height of ∼16.7 kcal mol^–1^ leading to formation of PC10 and then to ClC(O)CH_2_OH + CH_2_=C^•^H as products. Finally, it can directly release Cl atoms via C–Cl bond cleavage through transition state TS23 with a barrier height of 8.3 kcal mol^–1^ and then further proceed to form PC11, which goes on to produce CH_2_=CHC(O)CH_2_OH as a product. Once formed under tropospheric conditions, ^•^CH_2_OH rapidly combines with atmospheric ^3^O_2_ to form the corresponding peroxy radical which is reported to decompose to form formaldehyde and HO_2_ radical.^65^ Similarly, the CH_2_=C^•^H radical is expected to react with ^3^O_2_ leading to the formation of formaldehyde and HC(O) radical.^66^ The overall mechanism suggests that CP emissions into the atmosphere lead to the formation of products such as HOCH_2_C(OOH)(Cl)CH=CH_2_, HC(O)H, HO_2_ radical, ClC(O)CH=CH_2_, HOCH_2_C(O)Cl, HC(O) radical, Cl atom, and HOCH_2_C(O)CH=CH_2_. Therefore, the present work suggests that CP interacts with atmospheric hydroxyl radicals to yield products which, in subsequent reactions, lead to the formation of formaldehyde and a range of toxic chlorinated compounds in the environment.

## Supporting Information Available

Tables S1–S8: all the minima and transition state geometries optimized at the M06-2X level; vibrational frequencies, rotational constants, and imaginary frequencies computed at the M06-2X level; total electronic energies and zero-point energy, enthalpy and Gibbs free energy corrections calculated at various levels; enthalpies and Gibbs free energy changes for all possible paths; unimolecular rate coefficients and equilibrium constants for all possible paths; Figures S1 and S2: PES diagrams for the various abstraction paths associated with the CP + ^•^OH reaction and the isomerization reaction of IM1; Figures S3–S5: various possible unimolecular and bimolecular reaction channels for the RO_2_ radical adduct; Figure S6: PES profiles for alkoxy radical decomposition; Section S1: detailed explanation of the PES profiles and stationary point energies of the abstraction channels (PDF)
