# Supplementary material for: Atmospheric Chemistry of Chloroprene Initiated by OH Radicals: Combined Ab Initio/DFT Calculations and Kinetics Analysis
Source: J Phys Chem A. 2024 Oct 8;128(41):8983–95. doi: 10.1021/acs.jpca.4c05428 (PMC11492244; doi:10.1021/acs.jpca.4c05428)
Supplement: Supplementary file 1 — jp4c05428_si_001.pdf [file jp4c05428_si_001.pdf]

Atmospheric Chemistry of Chloroprene Initiated by OH Radicals: Combined *Ab Initio*/DFT Calculations and Kinetics Analysis

Parandaman Arathala and Rabi A. Musah*

University at Albany—State University of New York, Department of Chemistry, 1400 Washington Avenue, Albany, NY 12222, USA

*Address correspondence to: rmusah@albany.edu

This section contains : Tables S1−S8: all the minima and transition state geometries optimized at the M06-2X level; vibrational frequencies, rotational constants, and imaginary frequencies computed at the M06-2X level; total electronic energies and zero-point energy; enthalpy and Gibbs free energy corrections calculated at various levels; enthalpies and Gibbs free energy changes for all possible paths; unimolecular rate coefficients and equilibrium constants for all possible paths; Figures S1 and S2: PES diagrams for the various abstraction paths associated with the CP + $^{\bullet}$OH reaction and isomerization reaction of IM1; Figures S3-S5: various possible unimolecular and bimolecular reaction channels for the RO_2 radical adduct; Figure S6: PES profiles for alkoxy radical decomposition; Section S1: Detailed explanation of the PES profiles and stationary point energies for the abstraction channels.

Table S1: M06-2X/aug-cc-pVTZ level optimized geometries of various stationary points on the potential energy surfaces associated with the chloroprene + OH radical reaction.

***s-trans*-chloroprene**

C	-2.18090900	-0.69647300	0.00000000
C	-0.85409100	-0.69484100	-0.00003000
H	-2.71102800	0.24599200	0.00009800
H	-2.75145800	-1.61220400	-0.00006900
C	-0.04592000	0.52217500	0.00005800
C	1.27907400	0.58437500	0.00005800
H	-0.63293900	1.43397600	0.00012700
H	1.78386500	1.53953300	0.00012600
H	1.88851800	-0.30846200	-0.00000800
Cl	0.00358500	-2.21264000	-0.00019000

***gauche*-chloroprene**

C	0.05199300	1.63949100	0.10650400
C	-0.02785400	0.31841400	0.07791800
H	1.00314400	2.10770200	0.31748700
H	-0.81165500	2.26450200	-0.06177100
C	1.08436500	-0.62152300	0.26128100
C	2.30276200	-0.41335500	-0.21885200
H	0.85435000	-1.52833000	0.80774400
H	3.10020300	-1.12037500	-0.03918100
H	2.53192200	0.46160600	-0.81325600
Cl	-1.57233500	-0.45963700	-0.18387600

***s-cis*-chloroprene**

C	0.06596500	1.62286000	0.00016700
C	-0.03271800	0.30223700	0.00003000
H	1.03879200	2.09369700	0.00069800
H	-0.80881200	2.25405500	-0.00017200
C	1.06539900	-0.67789500	0.00028900
C	2.35724800	-0.37690400	-0.00032100
H	0.75790800	-1.71536100	0.00099800
H	3.10188600	-1.15964600	0.00003500
H	2.71680300	0.64352700	-0.00116300
Cl	-1.62060100	-0.43166100	-0.00042500

OH radical

O	0.00000000	0.00000000	-0.00249600

H	0.00000000	0.00000000	0.96938100

RC1

C	-1.51433800	0.60846700	-0.21492500
C	-0.19599400	0.62695600	-0.07593500
H	-1.97223800	1.29663400	-0.91258100
H	-2.15024700	-0.06416800	0.34141700
C	0.69631200	1.52127000	-0.80723200
C	2.01749200	1.57629200	-0.70210100
H	0.17735900	2.18476300	-1.48984600
H	2.58544800	2.27985800	-1.29323000
H	2.56306300	0.92822500	-0.03080700
Cl	0.56847600	-0.48008600	1.04892700
O	-2.34200800	-1.68725600	2.27882700
H	-1.40492400	-1.46185800	2.12804000

TS1

C	-2.17976200	-0.71888200	0.01997000
C	-0.83828500	-0.70498900	-0.01960000
H	-2.71641000	0.21771300	0.00277600
H	-2.74095000	-1.63683900	-0.04488200
C	-0.04609400	0.51283300	0.01416600
C	1.28044100	0.58430700	-0.01978800
H	-0.64054900	1.41694900	0.07750400
H	1.77924700	1.54203000	0.00969700
H	1.89524400	-0.30295200	-0.07772000
Cl	0.01547800	-2.22089900	-0.05502600
O	-2.35493500	-0.63704800	2.25485300
H	-1.60342100	-1.22187400	2.43990100

IM1

C	-1.59844700	0.26140600	0.20065400
C	-0.12688200	0.29414500	-0.04788400
H	-1.99098300	1.27204300	0.10054800
H	-2.08236500	-0.37136900	-0.55207500
C	0.60913300	1.42093900	-0.35882800
C	1.95389300	1.48052500	-0.61929800
H	0.03356200	2.33964700	-0.39395900
H	2.42577900	2.42224900	-0.85095900
H	2.57009500	0.59428600	-0.59732000
Cl	0.64477400	-1.25234500	0.07692700
O	-1.93515100	-0.17458200	1.50236800
H	-1.58279200	-1.06061900	1.62863100

TS2

C	-2.20149000	-0.58778900	-0.07463500
C	-0.87309200	-0.59044900	0.21597200
H	-2.69771200	0.35381300	-0.25442000
H	-2.77931600	-1.49869500	-0.07691400
C	-0.02245700	0.58978300	0.03795400
C	1.29153900	0.57818400	-0.11888700
H	-0.57567300	1.52130800	0.03973500
H	1.83469100	1.50187600	-0.25601300
H	1.85699400	-0.34295600	-0.11161900
Cl	-0.07476100	-2.13168900	0.36872400
O	-1.42000400	-0.14772400	2.12548900
H	-1.71453400	-1.02820700	2.40216500

IM2

C	-1.62032700	0.12233500	-0.74426100
C	-0.45827500	0.23723400	0.17807100
H	-2.03704400	1.02452000	-1.16494500
H	-2.11563500	-0.82497400	-0.88365700
C	0.44789600	1.36945200	-0.22275200
C	1.65723300	1.28402000	-0.74324500
H	-0.01802500	2.33473300	-0.05281300
H	2.19964000	2.17984700	-1.01080100
H	2.13935900	0.33160700	-0.91149300
Cl	0.41914800	-1.36755100	0.13345000
O	-0.85220100	0.49300700	1.49513400
H	-1.53947000	-0.13626600	1.74035100

RC2

C	-1.79835800	-0.39118800	-0.57401100
C	-0.61090500	-0.08946400	-0.06693600
H	-2.10226800	0.05981700	-1.50858200
H	-2.47397900	-1.07475100	-0.08359100
C	0.33440000	0.83562800	-0.68333800
C	1.56021600	1.11225500	-0.24868700
H	-0.05118600	1.32796700	-1.56853300
H	2.17731100	1.82978200	-0.76865600
H	1.97721600	0.63479600	0.62682400
Cl	-0.12524700	-0.80828900	1.45279000
O	0.23948100	2.68014400	1.48120700
H	0.06316600	1.82743900	1.91589200

TS3

C	-2.24901600	-0.72351900	-0.20088000
C	-0.95061100	-0.70213700	0.05718500
H	-2.77578600	0.21039100	-0.33963600
H	-2.80182400	-1.64741200	-0.27250000
C	-0.16366600	0.52853400	0.15607300
C	1.18496700	0.59948200	0.07795400
H	-0.75214100	1.43219700	0.07573800
H	1.67724400	1.55929200	0.06835300
H	1.79561200	-0.29199100	0.07208900
Cl	-0.08793400	-2.19739300	0.30778100
O	-0.05404700	0.79318200	2.21022800
H	0.39300200	-0.03595900	2.44067100

IM3

C	-1.80075400	-0.06367800	-0.89003800
C	-0.64600600	-0.04651200	-0.25325100
H	-2.27541900	0.87265100	-1.15032400
H	-2.29285300	-0.98586000	-1.16021300
C	0.12616500	1.18927600	0.14255100
C	1.41581800	1.29141400	-0.59466300
H	-0.51924500	2.04292900	-0.09430700
H	2.17553300	1.95643100	-0.21337400
H	1.53079500	0.85083000	-1.57252600
Cl	0.14602700	-1.53547100	0.20348500
O	0.31778000	1.25872500	1.54249400
H	0.90259500	0.53662100	1.79761000

TS4

C	-2.12528800	-0.70194200	0.04195500
C	-0.79623300	-0.67323900	0.04915800
H	-2.67332200	0.22917600	-0.00015100
H	-2.67700300	-1.62857700	0.07463600
C	-0.01327100	0.54903100	0.01097000
C	1.32827800	0.62751100	-0.01588000
H	-0.60340700	1.45675200	0.04685200
H	1.81698000	1.58699700	-0.07293700
H	1.94491300	-0.25403200	-0.10563200
Cl	0.09042800	-2.17521900	0.13343700
O	1.49380500	0.66755900	2.17949900
H	1.14542700	-0.22869000	2.31039600

IM4

C	-2.23497100	-0.13655800	-0.58342800
C	-0.89362700	0.01115000	-0.35285100
H	-2.84185900	0.74447800	-0.73204200
H	-2.70037100	-1.10767900	-0.62090300
C	-0.21098300	1.21161700	-0.28377300
C	1.25253300	1.33767600	-0.00006700
H	-0.80740500	2.10663100	-0.40398300
H	1.57991400	2.35604600	-0.19311800
H	1.81722700	0.66986200	-0.65742000
Cl	0.03967500	-1.46316400	-0.11346900
O	1.56988900	1.07781200	1.35979000
H	1.36805900	0.15493100	1.54147800

TS5

C	-2.10949700	-0.58917800	-0.08756100
C	-0.79235000	-0.63309300	0.00250300
H	-2.60122300	0.49592400	-0.34600600
H	-2.80941500	-1.40069400	0.04207900
C	0.08733900	0.52217500	-0.15779400
C	1.40988700	0.50268800	-0.05776700
H	-0.44229900	1.44198200	-0.37597900
H	1.98168600	1.40933600	-0.19314600
H	1.95163300	-0.40749300	0.15807000
Cl	-0.04306600	-2.18097200	0.31824500
O	-3.02919900	1.69376500	-0.27254700
H	-3.12090600	1.77760200	0.68898200

PC1

C	-1.38199100	-0.44773900	0.03401100
C	-0.07721100	-0.35213100	0.03224600
H	-2.42805800	1.94384300	-1.01520400
H	-2.12604900	-1.21966000	0.11558200
C	0.68000400	0.89153800	-0.10253500
C	2.00332600	0.98524400	-0.10622100
H	0.05682800	1.77249800	-0.20417700
H	2.48022100	1.94898100	-0.21126200
H	2.63787200	0.11602500	-0.00619100
Cl	0.84303900	-1.84738200	0.20675300
O	-2.30196800	2.58721800	-0.31277100
H	-2.43549400	2.08826500	0.49749300

CH_2=CHC(Cl)=C•H (IM5)

C	-1.37930000	-0.44438100	0.03363100
C	-0.07552500	-0.36154000	0.03325200
H	-2.13772000	-1.20165300	0.11358200
C	0.68126300	0.88151500	-0.10145600
C	2.00330500	0.98841200	-0.10655400
H	0.05662800	1.76085300	-0.20291400
H	2.47323700	1.95537800	-0.21198300
H	2.64325000	0.12325000	-0.00693500
Cl	0.85090100	-1.85446000	0.20758200

TS6

C	-2.25486500	-0.53262400	-0.03643900
C	-0.87766800	-0.56520100	-0.04518200
H	-2.63951500	0.48627200	0.02396700
H	-2.99786000	-1.64618300	-0.09341100
C	-0.02141900	0.58900400	0.01857000
C	1.31968400	0.58066900	0.00931000
H	-0.55297100	1.53096500	0.07858100
H	1.87509900	1.50598000	0.06148900
H	1.87859100	-0.34250100	-0.05028600
Cl	-0.10975900	-2.11618100	-0.13977700
O	-3.65128900	-2.66203700	-0.14237900
H	-3.04332600	-3.40914100	-0.18353900

PC2

C	-0.87555600	-0.09367000	-0.08943600
C	0.38622700	0.22857800	0.03776100
H	-1.78786000	0.45976300	-0.23816300
H	-2.82383400	3.18836600	0.47023800
C	0.88586200	1.60521200	-0.00237200
C	2.15519900	1.97148600	0.12151400
H	0.10332500	2.34103800	-0.14811800
H	2.42971700	3.01579000	0.07824900
H	2.94429500	1.24728700	0.26702200
Cl	1.56149000	-1.05291800	0.26988400
O	-2.44973800	2.78498600	-0.31646600
H	-2.82266400	3.26995800	-1.05617600

TS7

C	-2.17874200	-0.60155800	-0.01052600
C	-0.85444000	-0.66205800	0.01485800
H	-2.65666500	0.36604400	-0.08160400

H	-2.78626300	-1.49217200	0.03665900
C	0.01478200	0.50118900	-0.04540200
C	1.33055600	0.59641800	-0.02549300
H	-0.60882900	1.51622800	-0.13064700
H	1.81694600	1.56089700	-0.06662300
H	1.95257400	-0.28972700	0.02058200
Cl	-0.04302400	-2.20445800	0.12598900
O	-1.28550300	2.62467500	0.12508800
H	-1.12754000	2.67529400	1.08095000

PC3

C	-1.64855600	-0.19269200	0.00343700
C	-0.33719000	-0.38933300	-0.02474900
H	-2.02888600	0.82002200	0.02027100
H	-2.33727200	-1.02385200	0.01021500
C	0.65607700	0.64204100	-0.03628100
C	1.95805700	0.75900100	-0.06223500
H	-0.71888100	2.96791900	-0.67677100
H	2.44871600	1.72416500	-0.06382700
H	2.59016800	-0.12654200	-0.08336300
Cl	0.31624900	-2.02320700	-0.05111200
O	-1.25614200	3.18279900	0.09053000
H	-0.71556500	2.92137600	0.84085300

CH_2=C(Cl)$C^{\bullet}$=CH_2 (IM6)

C	-1.64536200	-0.19359700	0.00355300
C	-0.33430700	-0.38918800	-0.02471100
H	-2.02468000	0.81785900	0.01976500
H	-2.33807300	-1.02110600	0.01052000
C	0.65573100	0.64342200	-0.03620900
C	1.95687200	0.75907800	-0.06226700
H	2.44511100	1.72519500	-0.06366200
H	2.58992800	-0.12598400	-0.08331300
Cl	0.31214500	-2.02607500	-0.05132000

TS8

C	-2.16408100	-0.63652500	0.01123500
C	-0.83935700	-0.69781800	-0.00162200
H	-2.64999100	0.32885600	0.04463400
H	-2.77582400	-1.52528000	-0.01150800
C	0.02299000	0.48359800	0.02971700
C	1.34184000	0.47930000	0.00157600
H	-0.50806800	1.42903100	0.06898800

H	1.89127400	1.56285200	-0.00394400
H	2.00444900	-0.37478000	-0.02278200
Cl	-0.04222200	-2.24508100	-0.05816100
O	2.29817100	2.73305000	0.30556000
H	2.29400000	2.65174600	1.27133500

PC4

C	-2.26258700	-0.12236400	-0.05225500
C	-0.98384600	-0.47210600	-0.06357200
H	-2.52437500	0.92403900	0.02224000
H	-3.05318200	-0.85425400	-0.11440500
C	0.12101200	0.48841900	0.01899500
C	1.39538100	0.18838700	0.02074300
H	-0.18212800	1.53075700	0.08512700
H	2.12236000	3.94384100	0.10174500
H	2.01122800	-0.69330300	-0.02979300
Cl	-0.53971800	-2.15306000	-0.18132400
O	1.53306300	3.31005200	0.51566700
H	1.99666500	2.46688300	0.48189200

CH_2=C(Cl)CH=C$^{\bullet}$H (IM7)

C	-2.26203400	-0.12157500	-0.05314500
C	-0.98283100	-0.47045700	-0.06205700
H	-2.52615500	0.92449400	0.01864800
H	-3.05207000	-0.85388000	-0.11571100
C	0.12557700	0.48699200	0.02116000
C	1.40091100	0.19249200	0.01909800
H	-0.18830100	1.52563100	0.08944100
H	2.00714700	-0.69570300	-0.03425300
Cl	-0.54045900	-2.15147800	-0.17742500

RC3

C	-2.18660800	0.14765500	-0.27093900
C	-0.96860200	0.30944100	0.22753600
H	-2.58182300	0.89179800	-0.94860000
H	-2.79897800	-0.70503400	-0.02185800
C	-0.09598000	1.43618500	-0.08562100
C	1.10053700	1.67464100	0.44304400
H	-0.50107800	2.10627900	-0.83493400
H	1.67801200	2.53105300	0.12827500
H	1.53135700	1.03006500	1.19626700
Cl	-0.32391200	-0.90255100	1.31271800
O	1.88819200	-0.19964400	-1.31052800

H	1.49520200	-0.79629000	-0.64967300

TS9

C	-2.25429300	-0.66039500	0.00509800
C	-0.93126800	-0.74484200	0.05319500
H	-2.72166900	0.31040500	-0.08426500
H	-2.88301900	-1.53594200	0.05310700
C	-0.05396600	0.42040000	-0.00926900
C	1.26323200	0.48379200	0.01969200
H	-0.59900000	1.35873200	-0.08818000
H	1.83850900	1.39734100	-0.04613400
H	2.02937200	-0.47191300	0.11261100
Cl	-0.18872200	-2.31446400	0.20466800
O	2.92147700	-1.35347000	-0.00291500
H	2.33639400	-2.08694000	-0.24716200

PC5

C	-2.54560500	0.37921800	-0.07311800
C	-1.22976900	0.42062200	0.08138500
H	-3.07620900	1.28254100	-0.34059700
H	-3.10673900	-0.53243000	0.06096700
C	-0.41734300	1.62462800	-0.08666000
C	0.88153800	1.68809200	0.06398200
H	-0.98124000	2.51629700	-0.35962100
H	1.65404900	2.43211200	-0.01309900
H	2.19892600	-0.49151600	0.28149100
Cl	-0.36358700	-1.03166700	0.51519000
O	3.12359400	-0.56101100	0.02287000
H	3.11802400	-1.11424500	-0.76093900

RC4

C	-1.08922600	-0.06981300	-0.92340300
C	0.13442300	0.24705600	-0.52013600
H	-1.89940500	0.62228400	-0.74376700
H	-1.30434500	-1.00745900	-1.41248600
C	0.54372700	1.49794900	0.12400000
C	0.06832900	2.68399500	-0.23028300
H	1.27878900	1.40815300	0.91447400
H	0.37187300	3.58242300	0.28791700
H	-0.62901000	2.78895400	-1.05126900
Cl	1.43085500	-0.92042200	-0.69941300
O	-1.19229900	-1.26462400	1.44306100

H	-0.38908100	-1.72512700	1.14599600

TS10

C	-1.92968700	-0.85088900	-0.33657000
C	-0.71749300	-0.63991300	0.09046000
H	-2.59566000	-0.01098200	-0.51149400
H	-2.30443600	-1.84850700	-0.52211400
C	0.25287600	0.32632800	0.42685100
C	0.99256600	0.96939800	-0.49526200
H	0.43583900	0.49091300	1.48302000
H	1.74592000	1.68580000	-0.20178200
H	0.85076900	0.78161000	-1.55034100
Cl	0.39838200	-2.54189800	0.45152600
O	0.90174500	-4.26078800	0.60021300
H	0.31179500	-4.58796900	1.28976900

PC6

C	-2.32330400	0.93391800	-0.61795500
C	-1.20718800	1.44401100	-0.20432300
H	-3.12047500	1.55778000	-1.01035200
H	-2.49696300	-0.13764300	-0.58557000
C	-0.05221500	1.98359300	0.22175900
C	1.06922200	2.19048700	-0.57751200
H	-0.00350700	2.26453100	1.26915100
H	1.96525400	2.62273200	-0.16237800
H	1.06163900	1.91672500	-1.62128000
Cl	0.25753300	-1.12177400	0.48609700
O	0.59062400	-2.75649900	0.72528300
H	1.48207500	-2.86811000	0.37547900

$CH_2=^{\bullet}C-CH=CH_2$ (IM8)

C	-2.27352500	1.03476400	-1.01784800
C	-1.10753800	1.61813800	-0.89411800
H	-2.88836200	1.15445500	-1.90127600
H	-2.66913800	0.40094100	-0.22461800
C	-0.06021100	1.68793000	0.08529900
C	1.05865200	2.39015400	-0.06780600
H	-0.22190100	1.11905100	1.00038500
H	1.81767500	2.40977700	0.70101200
H	1.23681100	2.96092300	-0.96949000

TS11

C	-1.20314000	-0.01301100	-0.18801200

C	0.09710800	0.46245600	0.37099000
H	-1.51240200	0.66487600	-0.98323400
H	-1.09393400	-1.01895300	-0.60059700
C	0.62592400	1.80889400	0.31855300
C	1.24941500	1.48712300	-0.86431800
H	1.07583900	2.34041800	1.14178700
H	2.31397000	1.63562600	-1.05085900
H	0.68363800	1.11831300	-1.71304800
Cl	0.92103400	-0.71848700	1.37516800
O	-2.23993900	0.02248000	0.77961000
H	-1.99689800	-0.56340800	1.50276400

IM9

C	-1.23178100	-0.16558900	-0.75935800
C	0.13378100	0.27621800	-0.29482900
H	-1.60647300	0.57703700	-1.46368500
H	-1.13629800	-1.12444400	-1.27837600
C	0.48396800	1.66829700	-0.10979700
C	1.07531400	0.96759500	-1.25641900
H	0.91238000	2.20390000	0.71832800
H	2.12589300	0.70513600	-1.22399500
H	0.68518500	1.15624300	-2.25145000
Cl	0.84877300	-0.90495500	0.83975700
O	-2.16571600	-0.23833700	0.28755100
H	-1.83893000	-0.86231000	0.94322900

TS12

C	-1.60088600	-0.05643700	0.08870500
C	-0.13763500	0.19229800	-0.12569200
H	-2.12084800	-0.02132700	-0.87145400
H	-1.72163000	-1.06674700	0.49170400
C	0.44386000	1.47820500	-0.09967600
C	1.76199200	1.70694800	-0.23964700
H	-0.22451900	2.27332000	0.20649400
H	2.16720000	2.69832800	-0.09991000
H	2.44701300	0.91839600	-0.51563500
Cl	0.67894000	-1.10494300	-0.91373800
O	-2.18870800	0.91648800	0.91249300
H	-1.81736200	0.83117200	1.79607800
O	0.39750700	-0.31488000	1.86354600
O	1.51145400	0.08973100	2.14076100

RO_2 radical

C	-0.92370600	0.40500100	-1.08383300
C	0.08241000	0.03722600	0.01062700
H	-0.35324400	0.82552900	-1.91008800
H	-1.42784700	-0.49843000	-1.42248900
C	0.90505300	1.20612900	0.45581300
C	2.22235800	1.28850900	0.43705600
H	0.29249000	2.03433400	0.79282200
H	2.71583900	2.19322800	0.76272800
H	2.84250800	0.47038700	0.09878000
Cl	1.04386600	-1.34401700	-0.56876200
O	-1.82287000	1.38327300	-0.63392500
H	-2.48926500	0.95688200	-0.08683000
O	-0.61575000	-0.39261000	1.21616600
O	-1.68773600	-1.07805400	0.97364600

TS13

C	-1.03054300	0.30784300	-0.79210000
C	0.27205600	0.19099800	-0.29064900
H	-1.22699100	-0.18050800	-1.74396600
H	-1.56291300	-0.51532800	0.07603700
C	0.95613800	1.24159400	0.43891300
C	2.26085400	1.27098400	0.68868200
H	0.30463500	2.03938500	0.76886900
H	2.69435200	2.09791600	1.23208000
H	2.92372400	0.48420600	0.35686100
Cl	1.18893700	-1.13895200	-0.89797400
O	-1.62859400	1.55948000	-0.62022300
H	-2.55055800	1.44416000	-0.37941900
O	-0.38146600	-0.81948700	1.56382600
O	-1.56287600	-1.08951900	1.25533900

PC7

C	-1.20481700	0.73186500	-1.31994800
C	0.01440700	0.44348800	-0.86136500
H	-1.64559700	0.17127500	-2.13392700
H	0.37753400	-0.73049600	1.62733200
C	0.63400400	1.13021300	0.25631300
C	1.83219800	0.85471600	0.77296800
H	0.01742100	1.90755200	0.69051600
H	2.20640300	1.41498900	1.61752900
H	2.47120500	0.09146600	0.34885300
Cl	0.87537300	-0.86574100	-1.61641900

O	-1.92746200	1.74334900	-0.78994600
H	-2.82015700	1.73505800	-1.14050900
O	-1.41544700	-0.45740400	1.84267000
O	-0.38824300	-1.25765200	1.94324500

H_2C=CHC(Cl)=CHOH (P_1)

C	-1.21157400	0.72010900	-1.30682000
C	0.00470900	0.43287000	-0.84311600
H	-1.63750800	0.18314300	-2.14370600
C	0.63150000	1.11724100	0.27428400
C	1.83894200	0.85978400	0.76557400
H	0.02005200	1.89772700	0.71051300
H	2.22200500	1.42490800	1.60270300
H	2.46852900	0.08628900	0.34859100
Cl	0.88070100	-0.84326000	-1.63942300
O	-1.96315700	1.70135500	-0.74281100
H	-2.80122200	1.77806400	-1.20172500

TS14

C	-0.91485000	0.25870000	-1.18405200
C	0.18897700	-0.12057600	-0.23272600
H	-0.48732700	0.26263200	-2.19042900
H	-1.68663900	-0.51409800	-1.14800100
C	0.97719400	0.74955200	0.45998100
C	2.05369900	1.50407600	0.40080300
H	0.27500100	0.77098400	1.61115500
H	2.37254700	2.10192000	1.24387300
H	2.65839800	1.55407300	-0.49743700
Cl	0.63157800	-1.79008100	-0.30689300
O	-1.41912400	1.53918200	-0.94218800
H	-1.89422300	1.52613500	-0.10524500
O	-1.28189300	-0.28348000	1.40537700
O	-0.72576900	0.42869000	2.27914400

PC8

C	-0.90730700	0.14126200	-1.35741400
C	0.45532700	-0.13438000	-0.76075200
H	-0.87352200	-0.12445900	-2.41480300
H	-1.62748700	-0.52870600	-0.87327600
C	1.22964600	0.75043300	-0.20355900
C	1.96814100	1.66232300	0.35280700
H	-0.02582100	-0.08671200	1.46772200
H	1.88581700	1.88526500	1.41036400

H	2.68694100	2.22740500	-0.22771500
Cl	0.94260200	-1.81103100	-0.86160500
O	-1.27815900	1.48137500	-1.26224800
H	-1.40832700	1.68406900	-0.32796100
O	-1.50153900	0.97311500	1.67603300
O	-0.68271600	0.09012900	2.17638000

CH_2=C=CCl-CH_2OH (P_2)

C	-0.92158600	0.11544900	-1.34584100
C	0.44867600	-0.13695800	-0.77100300
H	-0.91304000	-0.15753500	-2.40134200
H	-1.63016800	-0.54936300	-0.83965100
C	1.20137700	0.75128000	-0.19880600
C	1.93179100	1.66624900	0.36485400
H	1.86547600	1.86124200	1.42839700
H	2.63584300	2.25263600	-0.21289700
Cl	0.96875600	-1.79813700	-0.91163000
O	-1.31209500	1.45882300	-1.27066200
H	-1.20135900	1.76987100	-0.36758200

TS15

C	-1.37285500	0.02929500	-0.97862100
C	-0.24251100	-0.10723300	0.03382700
H	-1.11763900	0.81871800	-1.68322100
H	-1.45042300	-0.91422900	-1.52437100
C	0.17761100	1.09968000	0.86724500
C	-0.69831300	2.05121300	1.30326400
H	1.23990400	1.28715900	0.92099200
H	-0.32603800	2.90469500	1.85085200
H	-1.76278200	1.94546900	1.16671800
Cl	1.20216900	-0.72278000	-0.86982200
O	-2.58615200	0.37681700	-0.36133600
H	-2.80213200	-0.30877900	0.27917100
O	-0.62909400	-0.97469800	1.04457400
O	-0.00504300	-0.31525600	2.13378900

$^{\bullet}CH_2$-cyc-C_2O_2HCl-CH_2OH (P_3)

C	-1.04806100	0.10109800	-1.15804700
C	0.13807500	-0.13840100	-0.24283700
H	-0.87391800	1.00911300	-1.73513400
H	-1.12355100	-0.74477200	-1.84727600
C	0.39769400	0.84535300	0.91376200
C	-0.29239500	2.13341000	0.97493900

H	1.46649200	0.93665600	1.10332300
H	0.27479300	3.04936400	1.00319100
H	-1.36948500	2.16108600	0.98772500
Cl	1.56400500	-0.52209900	-1.23183400
O	-2.22417300	0.28181900	-0.41463300
H	-2.32373800	-0.47195300	0.17740200
O	-0.15069100	-1.15179400	0.70893000
O	-0.16906800	-0.17533500	1.78815300

TS16

C	-1.27449100	1.05592300	-0.13222900
C	-0.10347600	0.08845300	-0.00190800
H	-1.27424600	1.72105100	0.72823600
H	-1.12248300	1.65138300	-1.03599500
C	-0.08673600	-0.84160100	1.17651600
C	-0.35893300	-2.14686500	0.86371400
H	0.39791700	-0.53180400	2.08901500
H	-0.02969500	-2.93835800	1.52527000
H	-1.14431900	-2.40578500	0.16905900
Cl	1.42011100	1.07428500	0.05290700
O	-2.49721900	0.36730700	-0.14162700
H	-2.53918800	-0.16712500	-0.93998600
O	-0.12510300	-0.74153600	-1.12260100
O	0.62798600	-1.87195900	-0.83244700

$OHCH_2$-cyc-$C_3O_2ClH_3$ (P_4)

C	-1.17520600	1.29401800	-0.10691800
C	-0.07986800	0.24448900	0.04795800
H	-1.12085100	1.97574300	0.73835400
H	-0.98176800	1.85621100	-1.02346000
C	-0.15393000	-0.66956900	1.19579400
C	0.02685800	-2.03961200	0.66199700
H	-0.30862600	-0.37255800	2.21696900
H	0.80769700	-2.61976100	1.15542600
H	-0.90989700	-2.61200800	0.66583200
Cl	1.52617800	1.15686700	0.09281700
O	-2.44640200	0.70007800	-0.09801800
H	-2.51586500	0.11696300	-0.85968500
O	-0.13756900	-0.58797400	-1.06463400
O	0.47091900	-1.82222200	-0.67620800

TS17

C	-0.80257100	0.63722600	-1.02903000

C	-0.00002100	0.29204600	0.34164300
H	-0.89478300	1.72904700	-1.04227500
H	-0.11076100	0.28014800	-1.80145100
C	1.12570900	1.26072100	0.50914200
C	2.39232700	1.01757700	0.22550900
H	0.79924500	2.23494000	0.85405300
H	3.13494600	1.79439500	0.33875400
H	2.72371900	0.04698500	-0.11640500
Cl	0.50251200	-1.41124000	0.27612500
O	-1.97152100	-0.01307000	-1.03983600
H	-2.22562700	-0.27618600	0.22230400
O	-0.90481200	0.53452500	1.35756900
O	-1.98141700	-0.31386600	1.28454100

CH_2=CHC(Cl)(OOH)$CH_2O^•$ (QOOH1)

C	-0.55382500	0.63834700	-1.29974700
C	0.12506700	0.33021900	0.04995400
H	-0.68559100	1.73591600	-1.35602200
H	0.09948000	0.35849200	-2.13396900
C	1.27710000	1.27731600	0.24338800
C	2.54853400	1.01927500	-0.00059700
H	0.95928300	2.25907400	0.57810300
H	3.29641300	1.78827800	0.13161300
H	2.87977900	0.04218200	-0.32344900
Cl	0.65018400	-1.38712500	0.02441100
O	-1.81926500	0.15850800	-1.44854500
H	-2.30183900	-0.19823900	0.45561500
O	-0.72751000	0.59076100	1.13094800
O	-1.76665000	-0.36400000	1.24704100

TS18

C	-0.95472300	0.33600000	-0.98394200
C	0.08739300	0.02305500	0.11953800
H	-0.61968800	0.25477500	-2.01127600
H	-1.63496300	-0.67833800	-0.57040500
C	0.88239800	1.20014500	0.58835600
C	2.18094200	1.38118300	0.42913000
H	0.27727900	1.95608800	1.07531500
H	2.65119900	2.28642000	0.78627900
H	2.80565500	0.64335100	-0.05345000
Cl	1.10393700	-1.31715300	-0.50499000
O	-1.67976400	1.47111300	-0.79103400
H	-1.98553600	1.50616100	0.12505100

O	-0.73179100	-0.40107000	1.17071500
O	-1.66053900	-1.27436400	0.59889800

$OHC^{\bullet}HC(Cl)(OOH)CHCH_2$ (QOOH2)

C	-0.81903200	0.58427400	-1.10715900
C	0.06348400	0.13198500	0.01829600
H	-0.44178200	0.66338000	-2.11349400
H	-2.05203200	-1.05799500	-0.02004000
C	0.88907700	1.26939000	0.56481000
C	2.20590300	1.36693200	0.54507100
H	0.28287500	2.07317700	0.96812700
H	2.69022400	2.25098800	0.93517300
H	2.83163200	0.58224000	0.14494300
Cl	1.06304700	-1.22710500	-0.57171400
O	-1.79972700	1.46229500	-0.78994200
H	-1.99643300	1.39814900	0.15442900
O	-0.74795000	-0.25333800	1.11011000
O	-1.57747800	-1.35262800	0.77440500

TS19

C	-0.99083000	0.32644800	-1.09359600
C	0.05590300	-0.03244000	-0.03416600
H	-0.47203700	0.58690100	-2.01465600
H	-1.59373100	-0.57022700	-1.26393700
C	0.88389500	1.14838900	0.39975200
C	1.96089200	1.74698800	-0.02282000
H	0.08388900	1.34218700	1.46049300
H	2.35285200	2.63006000	0.46449900
H	2.50676000	1.35793000	-0.87917500
Cl	1.03207300	-1.38340400	-0.64727100
O	-1.76607100	1.42977800	-0.72030900
H	-2.15031200	1.26682500	0.14781900
O	-0.53982200	-0.48144200	1.17602900
O	-0.85777200	0.68714400	1.87758100

$HOCH_2C(OOH)ClC^{\bullet}=CH_2$ (QOOH3)

C	-1.10605300	0.16379900	-1.03229400
C	0.06985700	-0.08183700	-0.07797800
H	-0.70265900	0.28579000	-2.03529800
H	-1.72916600	-0.73487500	-1.00526400
C	0.89043300	1.11281500	0.14359000
C	2.07273800	1.58001200	-0.13026900

H	-0.60976900	0.75977000	2.36336600
H	2.37264400	2.57934600	0.16129500
H	2.80003300	0.97102700	-0.66403400
Cl	1.06121600	-1.41526900	-0.77276300
O	-1.82528300	1.32899100	-0.73416700
H	-2.15833400	1.25418600	0.16501800
O	-0.35019400	-0.66241700	1.15871800
O	-1.21940100	0.26044900	1.80222600

TS20

C	-0.65530900	-0.60229300	1.08074000
C	0.06157700	0.15043600	-0.04830100
H	-1.43430900	0.04709500	1.47755600
H	-1.12847700	-1.47609700	0.62293900
C	0.83598000	1.36347300	0.42981300
C	2.12736900	1.29820500	0.59084500
H	0.24476200	2.25130500	0.63755600
H	2.88553800	1.97542800	0.95231000
H	2.44569100	-0.01645400	0.22667400
Cl	-1.17302500	0.63950300	-1.23698700
O	0.18476300	-0.94295200	2.14633000
H	0.92597900	-1.45101300	1.80099200
O	0.92307000	-0.68691500	-0.80460700
O	2.01123100	-1.04459400	-0.01360000

$HOCH_2C(OOH)ClCH=C^{•}H$ (QOOH4)

C	-0.94699300	-0.69734200	0.95477600
C	-0.17830500	0.12279200	-0.08740300
H	-1.81123200	-0.11614500	1.27087800
H	-1.29861500	-1.60454300	0.45566200
C	0.53019000	1.30467600	0.51707500
C	1.76791100	1.61993100	0.26545000
H	-0.07055400	1.89377600	1.20894000
H	2.46731100	2.38583800	0.55343600
H	2.33478900	-0.73615500	-0.01769700
Cl	-1.36833400	0.70876400	-1.29221300
O	-0.19484700	-0.96841900	2.10699400
H	0.58191100	-1.46912900	1.84031000
O	0.67467500	-0.67737900	-0.89067700
O	1.58810400	-1.35379600	-0.03626600

$RO^{•}$

C	-0.98024100	0.37517100	-1.09312100

C	0.08732700	0.01327400	-0.01804600
H	-0.45187600	0.74632600	-1.96797000
H	-1.51096100	-0.54349300	-1.34991500
C	0.89391900	1.24307500	0.38261000
C	2.20353300	1.27386500	0.54249100
H	0.26147000	2.10385300	0.55525300
H	2.69019000	2.19777200	0.82384500
H	2.82135600	0.40179900	0.38371800
Cl	1.12841100	-1.28821900	-0.76489000
O	-1.83064700	1.38361500	-0.63627900
H	-2.32897600	1.05387100	0.11751600
O	-0.51166200	-0.39546900	1.09285500

TS21

C	-0.94855400	0.46844400	-1.17749000
C	0.12123100	-0.10249900	0.39937000
H	-0.19756300	0.86536900	-1.84757800
H	-1.34680600	-0.51758500	-1.38946300
C	0.90632400	1.14972100	0.61377900
C	2.16340000	1.36159300	0.26225900
H	0.32042100	1.90414400	1.12472800
H	2.63769700	2.30641100	0.48858100
H	2.75021300	0.60589300	-0.23981100
Cl	1.07811900	-1.42955000	-0.45253100
O	-1.79879000	1.40141300	-0.73385500
H	-2.43164600	0.99876300	-0.12586600
O	-0.78220200	-0.44667700	1.14594400

PC9

C	-1.78656100	1.05238700	-1.66378900
C	0.29435200	-0.44970600	0.72249400
H	-1.97314300	1.78000200	-2.43636000
H	-1.62124100	0.01219300	-1.90700400
C	0.81906400	0.90573600	0.47927200
C	2.10582300	1.21771300	0.42472200
H	0.03681700	1.64162500	0.34315700
H	2.41118900	2.23878000	0.24370500
H	2.87908500	0.47404300	0.55439200
Cl	1.50286000	-1.73608100	0.93697100
O	-2.33889600	1.35620300	-0.46405600
H	-2.16792600	0.62682200	0.14648300
O	-0.86045600	-0.73089900	0.78646600

TS22

C	-0.97912000	0.38370200	-1.11429200
C	-0.18485600	-0.27891700	0.01087700
H	-0.31255000	0.86018400	-1.82723300
H	-1.50406000	-0.43617900	-1.61913600
C	0.99264600	1.42716300	0.43771400
C	2.28691000	1.35640700	0.51070900
H	0.21022400	2.15612100	0.57519300
H	2.86659300	2.24606400	0.74589600
H	2.82678400	0.43350200	0.33769800
Cl	1.06153000	-1.40976800	-0.66714800
O	-1.85334800	1.34158200	-0.59796400
H	-2.29724300	0.96034300	0.16816000
O	-0.64166700	-0.47476500	1.10759200

PC10

C	-1.20750700	0.29671800	-0.97362000
C	-0.64399000	-0.61749900	0.08412000
H	-0.38311400	0.82249000	-1.45444500
H	-1.68000000	-0.34976500	-1.72373000
C	1.09100800	1.82492400	0.66111400
C	2.36864900	1.58967200	0.74254800
H	0.41455500	2.64663100	0.82940600
H	3.07025600	2.35974900	1.05843500
H	2.78554200	0.61708500	0.50310500
Cl	0.70027700	-1.61115600	-0.51456600
O	-2.10186700	1.21448900	-0.42343500
H	-2.42160000	0.86394900	0.41598700
O	-1.05595200	-0.72315900	1.18582800

TS23

C	-0.59296400	0.22224100	-1.07947700
C	-0.10404900	0.10039700	0.35923700
H	0.24071100	0.18713200	-1.77644600
H	-1.26363100	-0.61370300	-1.27833100
C	1.05441700	0.90444200	0.81292400
C	1.63100400	1.83294900	0.06407900
H	1.35693400	0.71599100	1.83501100
H	2.44386000	2.43043100	0.45291500
H	1.29825800	2.04432600	-0.94320400
Cl	0.62589700	-1.92086000	0.35567600
O	-1.23125500	1.47067200	-1.24494000
H	-2.04240900	1.47534900	-0.72864100

O	-0.94493000	-0.28392900	1.23926600

PC11

C	-0.60021400	0.78050200	-1.17133700
C	-0.19398100	0.75885800	0.29078300
H	-0.58347000	1.81004400	-1.52949000
H	0.17937500	0.23302800	-1.72104900
C	0.97155000	1.56013500	0.73356000
C	1.70059200	2.32508900	-0.06836800
H	1.20394800	1.45769700	1.78597100
H	2.55401600	2.87146500	0.30901200
H	1.48261600	2.43316200	-1.12265900
Cl	-1.84049500	-2.22738500	0.67781300
O	-1.87395500	0.27674300	-1.40779700
H	-2.01396500	-0.51584400	-0.87487600
O	-0.78073500	0.07356100	1.10176000

CH_2=CHC(O)CH_2OH

C	-0.58067100	0.79315700	-1.18824400
C	-0.23049600	0.75998900	0.28081700
H	-0.73954700	1.84105200	-1.47271900
H	0.29403300	0.44261100	-1.75060900
C	0.95285800	1.55131100	0.70241600
C	1.33496500	1.58384400	1.97072500
H	1.48497400	2.09655700	-0.06809300
H	2.19417000	2.15546700	2.29239100
H	0.78011400	1.02699500	2.71546100
O	-1.70223800	0.02081000	-1.47302100
H	-1.99275600	-0.36121600	-0.63280300
O	-0.90433000	0.10959400	1.04608900

CH_2=CHC(O)Cl

C	0.06995400	-0.25813000	0.66263600
C	0.76344800	1.04031000	0.78034000
C	1.98179700	1.30107700	0.33256400
H	0.16183800	1.78550600	1.28426700
H	2.41374400	2.28366800	0.46184900
H	2.57380700	0.54928100	-0.16942800
Cl	0.99295900	-1.55618800	-0.15760700
O	-1.01951800	-0.48037400	1.06443300

HOCH_2C(O)Cl

C	-1.20132800	0.29838100	-0.97835300

C	-0.65802100	-0.61590800	0.08793300
H	-0.37478600	0.92604700	-1.32429200
H	-1.51181800	-0.32745500	-1.82019300
Cl	0.68868200	-1.61338900	-0.48385500
O	-2.25933500	1.07002900	-0.50284900
H	-2.41429800	0.84401400	0.42177500
O	-1.06284900	-0.68565200	1.19597300

$^{\bullet}CH_2OH$

C	-1.41633100	0.66241700	-1.54299200
H	-1.07553400	1.24560700	-2.38163300
H	-1.65987600	-0.38406100	-1.65438400
O	-2.06887300	1.38972500	-0.59735500
H	-2.41644800	0.80997800	0.08376400

$CH_2=C^{\bullet}H$

C	1.08634400	1.81774000	0.65962900
C	2.36510000	1.58908100	0.74330600
H	0.42423800	2.65083300	0.83066800
H	3.06576800	2.36075900	1.05794600
H	2.78856000	0.61964700	0.50306000

Table S2: The harmonic vibrational frequencies obtained at the M06-2X/aug-cc-pVTZ level for all the stationary points associated with the chloroprene + OH radical reaction.

s-trans-chloroprene	151 256 393 422 531 648 677 773 912<br>937 984 1022 1042 1250 1323 1414 1458 1681<br>1738 3177 3186 3189 3273 3284
RC1	2 79 116 144 172 263 367 410 422<br>531 644 681 776 917 955 983 1023 1043<br>1247 1324 1423 1463 1682 1739 3176 3180 3189<br>3273 3275 3704
TS1	-289 67 86 150 184 257 392 432 533<br>631 660 717 799 917 943 985 1022 1043<br>1254 1321 1406 1459 1611 1702 3182 3192 3200<br>3275 3300 3784
IM1	70 168 239 266 315 353 457 553 605<br>657 843 933 970 1004 1079 1154 1203 1222<br>1325 1362 1418 1445 1494 1517 3024 3129 3177<br>3189 3288 3861
TS2	-491 109 173 205 256 278 351 390 524<br>614 637 717 786 880 914 986 1018 1039<br>1251 1315 1394 1456 1564 1734 3183 3191 3197

	3274 3298 3799
IM2	73 83 248 306 328 341 410 419 481<br>505 644 716 848 996 999 1029 1075 1142<br>1170 1321 1369 1449 1454 1748 3171 3178 3186<br>3270 3297 3839
RC2	43 60 130 171 243 261 391 429 460<br>530 643 684 776 911 947 989 1017 1041<br>1245 1321 1415 1459 1676 1729 3183 3186 3192<br>3277 3282 3743
TS3	-430 63 139 192 258 285 390 478 534<br>643 660 733 774 908 937 950 1014 1043<br>1245 1305 1406 1456 1606 1720 3185 3188 3213<br>3282 3290 3784
IM3	79 147 216 228 334 350 438 472 619<br>633 640 738 877 953 959 1059 1140 1226<br>1244 1304 1398 1438 1449 1734 3030 3178 3180<br>3273 3292 3836
TS4	-342 55 79 187 238 267 391 480 533<br>645 673 692 792 916 941 984 997 1041<br>1252 1312 1408 1457 1627 1680 3185 3195 3203<br>3284 3295 3779
IM4	90 132 234 359 377 411 496 553 564<br>636 751 815 888 994 1062 1110 1191 1219<br>1297 1367 1406 1425 1498 1509 3046 3141 3186<br>3201 3296 3859
TS5	-1441 98 108 134 180 260 310 422 523<br>528 650 748 765 844 899 989 1013 1038<br>1192 1246 1266 1335 1448 1666 1731 3183 3198<br>3247 3273 3799
PC1	23 77 102 112 140 210 238 256 365<br>420 520 633 694 743 823 980 988 1047<br>1170 1334 1445 1618 1655 1723 3182 3192 3275<br>3293 3851 3947
H_2C=CH-CCl=$^{\bullet}$CH (IM5)	146 252 350 410 520 627 705 738 806<br>975 982 1023 1167 1321 1444 1659 1725 3183<br>3195 3276 3300
TS6	-4578 25 72 155 260 276 343 424 500<br>564 619 680 690 943 972 1003 1023 1100<br>1252 1269 1325 1406 1490 1614 1635 3121 3182<br>3196 3278 3845
PC2	14 49 95 102 135 161 181 248 370<br>427 524 614 703 760 839 978 988 1045<br>1179 1326 1442 1619 1650 1717 3179 3193 3270<br>3273 3861 3963
TS7	-1096 61 97 166 200 243 383 398 494<br>625 664 706 764 859 918 962 966 1024

	1183 1228 1287 1412 1440 1683 1739 3162 3185 3256 3286 3791
PC3	26 71 99 103 124 186 190 240 368 385 483 630 632 761 888 925 980 1008 1171 1403 1434 1619 1680 1770 3112 3175 3214 3280 3850 3944
$H_2C=C^{\bullet}-CCl=CH_2$ (IM6)	122 186 364 374 476 622 628 742 884 926 950 1004 1165 1399 1429 1684 1775 3110 3189 3216 3287
TS8	-1398 81 93 143 177 207 391 423 497 603 652 710 808 860 907 946 971 1012 1224 1237 1286 1295 1429 1677 1724 3175 3186 3227 3282 3803
PC4	30 57 92 121 134 178 258 276 393 473 511 656 696 760 877 921 946 955 1212 1274 1425 1616 1662 1706 3145 3184 3276 3281 3829 3946
$H^{\bullet}C=CH-CCl=CH_2$ (IM7)	148 242 392 465 508 654 693 770 870 908 939 953 1209 1262 1425 1663 1706 3142 3184 3279 3281
RC3	43 60 126 163 231 261 390 427 474 531 642 682 774 910 945 985 1016 1041 1245 1322 1414 1460 1677 1730 3184 3187 3193 3278 3283 3741
TS9	-1623 36 100 149 217 247 395 424 517 576 647 679 753 797 912 938 950 976 1068 1250 1301 1425 1460 1676 1732 3135 3187 3222 3284 3800
PC5	18 43 72 98 149 167 233 356 395 457 525 652 679 766 797 893 942 963 1231 1250 1427 1623 1667 1716 3109 3185 3283 3293 3839 3946
TS10	-415 67 76 106 134 201 226 267 456 518 624 667 882 933 954 990 999 1085 1119 1304 1403 1431 1579 1809 3132 3173 3179 3228 3273 3846
PC6	50 68 77 79 98 106 216 253 511 535 579 753 787 914 918 945 983 1079 1171 1265 1367 1447 1489 1925 3115 3162 3187 3187 3292 3847
$H_2C=CH-C^{\bullet}=CH_2$ (IM8)	64 231 409 478 741 878 912 977 980 1011 1159 1303 1406 1449 1684 1756 3092 3113 3175 3204 3266
TS11	-1125 55 146 195 291 309 364 462 587 660 796 927 979 988 1032 1079 1186 1221 1323 1366 1376 1420 1495 1533 3056 3079 3121

	3175 3241 3860
IM9	119 205 291 305 335 360 487 606 617 845 898 933 973 1029 1063 1094 1120 1156 1231 1365 1379 1427 1471 1498 3045 3115 3119 3200 3274 3853
TS12	-432 82 102 134 180 191 245 265 308 365 407 413 550 641 719 953 963 970 1011 1116 1169 1206 1264 1332 1383 1407 1445 1485 1498 1618 3046 3091 3185 3202 3279 3858
RO_2 radical	72 84 139 201 247 319 344 345 391 415 471 566 687 770 854 912 1010 1037 1050 1107 1148 1179 1269 1285 1325 1395 1405 1454 1497 1746 3099 3153 3179 3195 3270 3861
TS13	-1313 98 117 127 191 214 222 241 291 381 444 501 569 665 745 755 969 992 1005 1033 1127 1206 1254 1300 1315 1354 1407 1452 1557 1623 1709 3134 3184 3218 3274 3884
PC7	30 43 57 97 172 203 218 224 271 309 407 456 467 543 739 756 914 964 989 1043 1168 1202 1269 1288 1326 1383 1463 1498 1692 1752 3176 3204 3210 3268 3520 3907
H_2C=CHC(Cl)=CHOH (P_1)	137 211 213 270 302 404 454 541 737 740 897 967 988 1031 1162 1193 1285 1326 1383 1462 1704 1759 3182 3199 3214 3272 3914
TS14	-1131 77 124 137 165 194 227 293 351 398 462 481 555 637 682 775 970 981 1029 1088 1096 1156 1209 1311 1385 1398 1419 1488 1516 1685 1884 3053 3095 3150 3237 3855
PC8	40 59 65 126 136 153 196 235 273 397 415 460 509 529 616 728 938 992 1000 1020 1157 1237 1283 1288 1389 1423 1479 1492 1502 2086 3032 3108 3149 3230 3520 3817
CH_2=C=CClCH_2OH (P_2)	108 136 221 236 278 405 457 533 621 724 937 996 1015 1023 1143 1225 1296 1361 1418 1486 1509 2099 3035 3111 3151 3230 3862
TS15	-764 72 165 216 218 241 337 360 389 420 446 508 667 781 865 920 930 952 1024 1070 1111 1158 1245 1278 1309 1379 1421 1441 1495 1551 3069 3143 3185 3227 3294 3850
$^{\bullet}CH_2$-cyc-C_2O_2HCl-CH_2OH (P_3)	109 171 197 216 235 252 341 396 426 436 482 593 647 816 888 921 991 1011 1061 1103 1146 1174 1229 1265 1271 1371 1391 1428 1470 1493 3056 3109 3129 3194 3309 3840
TS16	-763 114 130 201 221 320 340 384 407 450 504 660 714 775 886 911 928 992 1016 1042 1110 1150 1199 1249 1285 1374 1412

	1419 1497 1545 3068 3151 3168 3239 3257 3866
$OHCH_2$-cyc-$C_3O_2ClH_3$ (P_4)	93 129 190 199 229 340 379 391 422<br>661 730 762 910 919 953 985 991 1066<br>1115 1153 1183 1206 1272 1353 1361 1388 1413<br>1489 1501 3001 3071 3102 3153 3291 3865
TS17	-1171 78 151 217 245 277 333 432 446<br>515 572 600 617 684 769 839 948 999<br>1011 1030 1079 1171 1203 1205 1242 1320 1327<br>1451 1498 1739 1817 2999 3068 3179 3194 3271
QOOH1	75 94 187 228 247 279 330 351 415<br>451 479 567 670 688 825 932 1008 1026<br>1030 1053 1092 1147 1159 1229 1322 1336 1368<br>1451 1472 1744 2930 3045 3175 3186 3269 3749
TS18	-1938 66 142 209 216 254 324 352 445<br>460 507 588 653 681 824 900 994 1008<br>1017 1030 1058 1094 1182 1192 1231 1317 1331<br>1420 1449 1739 1780 3180 3192 3193 3274 3800
QOOH2	76 117 162 174 190 250 291 323 373<br>430 470 514 569 625 699 796 930 998<br>1005 1019 1069 1111 1162 1204 1316 1332 1432<br>1447 1453 1734 3175 3188 3257 3272 3723 3793
TS19	-2167 102 125 194 205 255 294 331 391<br>419 463 520 592 657 749 888 937 951<br>998 1025 1044 1085 1146 1163 1253 1375 1403<br>1429 1497 1745 1815 3056 3124 3137 3227 3840
QOOH3	79 123 143 190 227 251 292 323 389<br>398 419 470 480 644 746 853 944 956<br>1000 1024 1109 1149 1171 1262 1380 1402 1416<br>1436 1493 1788 3059 3107 3144 3207 3790 3863
TS20	-1691 115 158 221 239 306 336 396 430<br>450 484 545 590 700 771 849 885 961<br>978 1044 1086 1120 1148 1169 1235 1273 1382<br>1434 1496 1601 1717 3057 3132 3148 3256 3849
QOOH4	93 126 174 207 245 278 301 332 363<br>425 453 490 576 657 745 855 887 922<br>984 1029 1120 1158 1177 1263 1279 1384 1410<br>1436 1496 1704 3061 3115 3138 3277 3754 3856

Table S3: Rotational constants obtained at the M06-2X/aug-cc-pVTZ level for various stationary points associated with the chloroprene + OH radical reaction.

Species	A	B	C
s-trans-chloroprene	5.47	3.76	2.23
RC1	5.32	1.38	1.10

TS1	3.07	2.16	1.54
IM1	4.05	2.17	1.51
TS2	2.98	2.71	1.93
IM2	3.26	2.80	2.08
RC2	3.12	2.01	1.70
TS3	3.55	2.13	1.79
IM3	3.72	2.32	1.95
TS4	3.69	1.91	1.55
IM4	4.26	2.16	1.57
TS5	3.77	1.67	1.16
PC1	3.96	1.40	1.04
$H_2C=CH-CCl={}^{\bullet}CH$ (IM5)	5.91	3.73	2.29
TS6	5.02	1.51	1.16
PC2	4.10	1.28	0.98
TS7	4.44	1.64	1.21
PC3	4.10	1.41	1.05
$H_2C=C^{\bullet}-CCl=CH_2$ (IM6)	5.68	3.63	2.21
TS8	5.36	1.20	0.99
PC4	5.33	1.12	0.93
$H^{\bullet}C=CH-CCl=CH_2$ (IM7)	5.47	4.07	2.33
RC3	3.13	2.02	1.70
TS9	3.86	1.98	1.31
PC5	3.87	1.61	1.16
TS10	4.91	1.46	1.18
PC6	4.13	1.34	1.06
$H_2C=CH-C^{\bullet}=CH_2$ (IM8)	55.21	4.36	4.04
TS11	3.27	2.60	1.66
IM9	3.44	2.85	1.86
TS12	1.73	1.50	1.26
RO_2 radical	2.05	1.53	1.23
TS13	1.80	1.39	1.22
PC7	1.51	1.31	1.01
$H_2C=CHC(Cl)=CHOH$ (P_1)	3.69	2.37	1.44
TS14	1.61	1.36	1.34
PC8	1.66	1.09	1.06
$CH_2=C=CClCH_2OH$ (P_2)	3.31	2.33	1.40
TS15	2.10	1.60	1.26
${}^{\bullet}CH_2$-cyc-$C_2O_2HCl-CH_2OH$ (P_3)	2.26	1.52	1.32
TS16	1.96	1.78	1.23
$OHCH_2$-cyc-$C_3O_2ClH_3$ (P_4)	1.90	1.89	1.21
TS17	2.40	1.44	1.32
QOOH1	2.21	1.41	1.32
TS18	2.03	1.62	1.21
QOOH2	1.98	1.58	1.21
TS19	1.97	1.46	1.34

QOOH3	1.97	1.39	1.29
TS20	2.23	1.49	1.29
QOOH4	2.02	1.49	1.30

Table S4. Imaginary frequencies of transition states optimized at the M06-2X/aug-cc-pVTZ level.

Transition state	Imaginary frequency
TS1	289
TS2	491
TS3	430
TS4	342
TS5	1441
TS6	4578
TS7	1096
TS8	1398
TS9	1623
TS10	415
TS11	1125
TS12	432
TS13	1313
TS14	1131
TS15	764
TS16	763
TS17	1171
TS18	1938
TS19	2167
TS20	1691
TS21	273
TS22	437
TS23	322

Table S5: Calculated total electronic energies for all the stationary points involved in the CP + OH radical reaction at the M06-2X/aug-cc-pVTZ and CCSD(T)/aug-cc-pVTZ levels of theory. Zero-point energy (ZPE) corrections and thermal corrections to Gibbs free energies and enthalpies are given at the M06-2X level.

Species	M06-2X	ZPE(M06-2X)	CCSD(T)	Thermal correction to enthalpy	Thermal correction to free energy
s-trans-chloroprene	-615.581719	0.076821	-614.847792	0.083113	0.047925
$^{\bullet}$OH	-75.733810	0.008590	-75.645584	0.011895	-0.008331
RC1	-691.320851	0.087041	-690.498812	0.096681	0.049353
TS1	-691.322565	0.087716	-690.498671	0.096259	0.054444
IM1	-691.387948	0.091851	-690.560818	0.099809	0.059772
TS2	-691.313856	0.087785	-690.492175	0.095989	0.055880
IM2	-691.362759	0.089073	-690.537518	0.097631	0.056531
RC2	-691.323200	0.087557	-690.498944	0.096896	0.053096
TS3	-691.317720	0.088124	-690.495287	0.096326	0.055686
IM3	-691.362958	0.089901	-690.536062	0.098207	0.057725
TS4	-691.321570	0.087913	-690.498144	0.096301	0.054771
IM4	-691.382346	0.091388	-690.555614	0.099213	0.059597
TS5	-691.305668	0.082228	-690.481840	0.090918	0.049032
PC1	-691.330558	0.086697	-690.502856	0.097197	0.050121
$H_2C{=}CH{-}CCl{=}^{\bullet}CH$ (IM5)	-614.895449	0.063343	-614.157015	0.069710	0.033769
H_2O	-76.430105	0.021547	-76.342289	0.025327	0.003259
TS6	-691.255007	0.082608	-690.426384	0.091307	0.048408
PC2	-691.328181	0.086357	-690.501978	0.097136	0.048458
TS7	-691.308664	0.082428	-690.485300	0.091063	0.049127
PC3	-691.336515	0.085998	-690.507999	0.096770	0.049035
$H_2C{=}C^{\bullet}{-}CCl{=}CH_2$ (IM6)	-614.900831	0.062722	-614.161543	0.069314	0.032772
TS8	-691.304942	0.082073	-690.479642	0.090783	0.048572
PC4	-691.330697	0.086330	-690.503533	0.096798	0.049700
$H^{\bullet}C{=}CH{-}CCl{=}CH_2$ (IM7)	-614.895508	0.063089	-614.156721	0.069376	0.033594
RC3	-691.323188	0.087523	-690.498942	0.096890	0.052998
TS9	-691.303457	0.081687	-690.477840	0.090403	0.048011

PC5	-691.331584	0.086056	-690.503827	0.096626	0.048582
TS10	-691.263358	0.085798	-690.438840	0.095143	0.051284
PC6	-691.273862	0.085219	-690.445847	0.095691	0.048231
H_2C=CH-C$^{\bullet}$=CH_2 (IM8)	-155.288172	0.071277	-155.032482	0.077285	0.043403
HOCl	-535.967713	0.013500	-535.408682	0.017366	-0.009412
TS11	-691.300507	0.089584	-690.473273	0.097484	0.057250
IM9	-691.345654	0.092079	-690.513329	0.099611	0.060906
$^3O_2$	-150.324795	0.003997	-150.140247	0.007303	-0.015943
TS12	-841.711452	0.098148	-840.699282	0.108292	0.062989
RO_2 radical	-841.744262	0.101337	-840.734407	0.111096	0.066588
TS13	-841.687604	0.094679	-840.681413	0.104719	0.059754
PC7	-841.728951	0.098476	-840.716894	0.110080	0.059673
H_2C=CHC(Cl)=CHOH (P_1)	-690.810397	0.081902	-689.980913	0.089505	0.051116
HO_2 radical	-150.908094	0.014567	-150.726056	0.018363	-0.007577
TS14	-841.670812	0.094693	-840.662026	0.104685	0.059688
PC8	-841.705033	0.098139	-840.692292	0.109696	0.059865
CH_2=C=CClCH_2OH (P_2)	-690.783811	0.081145	-689.953167	0.089034	0.049746
TS15	-841.697957	0.099650	-840.692324	0.108811	0.065896
$^{\bullet}CH_2$-cyc-C_2O_2HCl-CH_2OH (P_3)	-841.721848	0.100626	-840.713172	0.110024	0.066973
TS16	-841.700627	0.100205	-840.693975	0.109040	0.066884
OHCH_2-cyc-$C_3O_2ClH_3$ (P_4)	-841.751462	0.101543	-840.739683	0.110834	0.067637
TS17	-841.701544	0.094609	-840.690269	0.103466	0.061207
QOOH1	-841.708669	0.099349	-840.698998	0.109030	0.064951
TS18	-841.693098	0.095923	-840.684677	0.105042	0.062065
QOOH2	-841.727818	0.099518	-840.716509	0.109770	0.064599
TS19	-841.676758	0.094401	-840.667938	0.103830	0.060493
QOOH3	-841.707444	0.099623	-840.695341	0.109906	0.064725
TS20	-841.689045	0.094681	-840.679332	0.103643	0.061489
QOOH4	-841.704519	0.099728	-840.692707	0.109715	0.065372
RO$^{\bullet}$	-766.585332	0.095857	-765.6667483	0.104821	0.062226
TS21	-766.581870	0.094378	-765.6606069	0.103292	0.061007
$^{\bullet}CH_2$OH	-115.056126	0.037583	-114.8987699	0.041889	0.014595

CH_2=CHC(=O)Cl	-651.527395	0.053343	-650.7660878	0.059414	0.024373
TS22	-766.571026	0.095881	-765.6535564	0.104596	0.062666
PC10	-766.591958	0.096319	-765.6740014	0.106204	0.059528
$^{\bullet}$Cl	-460.141208	0.000000	-459.6762157	0.002360	-0.015677
CH_2=CHC(O)CH_2OH	-306.444414	0.095764	-305.9930082	0.103221	0.065499
TS23	-766.557087	0.092220	-765.6364324	0.101599	0.058130
PC11	-766.568196	0.091224	-765.645929	0.102529	0.052471
CH_2=C$^{\bullet}$H	-77.889259	0.036965	-77.7569295	0.040988	0.014498
HOCH_2C(O)Cl	-688.672233	0.052944	-687.8841015	0.059492	0.023285

Table S6: Enthalpies and Gibbs free energy changes (in kcal mol^{-1}) calculated for the H-abstraction and OH addition paths associated with the reaction of CP + OH radical at the CCSD(T)/aug-cc-pVTZ//M06-2X/aug-cc-pVTZ level.

Path	Stationary point	ΔH (298 K)[a]	ΔG (298 K)[a]
R1	CP + $^{\bullet}$OH	0.0	0.0
	RC1	-2.4	2.7
	TS1	-2.5	6.0
	IM1	-39.3	-29.7
R2	TS2	1.4	11.0
	IM2	-26.1	-17.1
R3	RC2	-2.3	5.0
	TS3	-0.4	8.9
	IM3	-24.8	-15.4
R4	TS4	-2.2	6.5
	IM4	-36.4	-26.5
R5	TS5	4.7	13.2
	PC1	-4.6	0.7
	IM5 + H_2O	-3.7	-5.3
	TS6	39.7	47.6
	PC2	27.9	33.6
R6	TS7	2.6	11.1
	PC3	-8.1	-3.3
	IM6 + H_2O	-6.8	0.0
R7	TS8	6.0	14.3
	PC4	-5.3	0.0
	IM7 + H_2O	-3.7	-5.3
	RC3	-2.3	4.9
	TS9	6.9	15.0
	PC5	-5.5	-0.9
R8	RC4	0.3	6.9
	TS10	34.3	41.6
	PC6	30.3	35.2
	IM8 + HOCl	32.5	29.2

[a]Energies computed at the CCSD(T)/aug-cc-pVTZ level and the thermodynamic corrections computed at the M06-2X/aug-cc-pVTZ level.

Table S7: Equilibrium constants (in cm^3 $molecule^{-1}$) for the formation of pre-reactive complexes from the CP + OH radical reactants calculated between 200 and 300 K temperature.

T (K)	RC1	RC2	RC3	RC4
200	2.18×10^{-21}	4.49×10^{-23}	4.97×10^{-23}	1.89×10^{-25}
210	1.68×10^{-21}	3.51×10^{-23}	3.88×10^{-23}	2.02×10^{-25}
220	1.34×10^{-21}	2.81×10^{-23}	3.11×10^{-23}	2.15×10^{-25}
230	1.09×10^{-21}	2.31×10^{-23}	2.55×10^{-23}	2.29×10^{-25}
240	9.06×10^{-22}	1.93×10^{-23}	2.14×10^{-23}	2.44×10^{-25}
250	7.67×10^{-22}	1.64×10^{-23}	1.82×10^{-23}	2.58×10^{-25}
260	6.60×10^{-22}	1.42×10^{-23}	1.57×10^{-23}	2.74×10^{-25}
270	5.75×10^{-22}	1.25×10^{-23}	1.38×10^{-23}	2.90×10^{-25}
280	5.08×10^{-22}	1.10×10^{-23}	1.22×10^{-23}	3.06×10^{-25}
290	4.54×10^{-22}	9.89×10^{-24}	1.09×10^{-23}	3.23×10^{-25}
298.15	4.17×10^{-22}	9.11×10^{-24}	1.01×10^{-23}	3.37×10^{-25}
300	4.09×10^{-22}	8.95×10^{-24}	9.89×10^{-24}	3.40×10^{-25}
400	2.12×10^{-22}	4.72×10^{-24}	5.22×10^{-24}	5.43×10^{-25}

Table S8: Unimolecular rate coefficients (in s^{-1}) for the transformation of pre-reactive complexes to post-reactive complexes via their corresponding transition states in various addition and abstraction paths calculated between 200 and 400 K temperature.

T (K)	TS1	TS2	TS3	TS4	TS5	TS6	TS7	TS8	TS9	TS10
200	1.77×10^{11}	1.77×10^{6}	1.18×10^{9}	2.62×10^{11}	2.00×10^{4}	1.81×10^{-9}	4.22×10^{10}	3.95×10^{4}	1.30×10^{4}	1.26×10^{-25}
210	1.92×10^{11}	2.90×10^{6}	1.60×10^{9}	2.88×10^{11}	3.88×10^{4}	1.74×10^{-9}	4.33×10^{10}	8.08×10^{4}	2.67×10^{4}	7.62×10^{-24}
220	2.08×10^{11}	4.57×10^{6}	2.11×10^{9}	3.13×10^{11}	7.17×10^{4}	1.67×10^{-9}	4.44×10^{10}	1.56×10^{5}	5.23×10^{4}	3.18×10^{-22}
230	2.23×10^{11}	6.92×10^{6}	2.71×10^{9}	3.38×10^{11}	1.27×10^{5}	1.61×10^{-9}	4.55×10^{10}	2.87×10^{5}	9.76×10^{4}	9.62×10^{-21}
240	2.39×10^{11}	1.01×10^{7}	3.42×10^{9}	3.62×10^{11}	2.15×10^{5}	1.55×10^{-9}	4.66×10^{10}	5.05×10^{5}	1.74×10^{5}	2.20×10^{-19}
250	2.54×10^{11}	1.44×10^{7}	4.23×10^{9}	3.86×10^{11}	3.51×10^{5}	1.50×10^{-9}	4.77×10^{10}	8.54×10^{5}	3.00×10^{5}	3.91×10^{-18}
260	2.68×10^{11}	2.00×10^{7}	5.15×10^{9}	4.09×10^{11}	5.55×10^{5}	1.45×10^{-9}	4.88×10^{10}	1.39×10^{6}	4.97×10^{5}	5.59×10^{-17}
270	2.82×10^{11}	2.70×10^{7}	6.17×10^{9}	4.33×10^{11}	8.52×10^{5}	1.40×10^{-9}	4.99×10^{10}	2.19×10^{6}	7.98×10^{5}	6.57×10^{-16}
280	2.96×10^{11}	3.57×10^{7}	7.29×10^{9}	4.55×10^{11}	1.27×10^{6}	1.36×10^{-9}	5.10×10^{10}	3.36×10^{6}	1.24×10^{6}	6.48×10^{-15}
290	3.10×10^{11}	4.64×10^{7}	8.52×10^{9}	4.77×10^{11}	1.85×10^{6}	1.33×10^{-9}	5.21×10^{10}	5.00×10^{6}	1.88×10^{6}	5.47×10^{-14}
298.15	3.20×10^{11}	5.67×10^{7}	9.59×10^{9}	4.95×10^{11}	2.47×10^{6}	1.30×10^{-9}	5.30×10^{10}	6.80×10^{6}	2.60×10^{6}	2.80×10^{-13}
300	3.23×10^{11}	5.93×10^{7}	9.85×10^{9}	4.99×10^{11}	2.64×10^{6}	1.29×10^{-9}	5.32×10^{10}	7.27×10^{6}	2.79×10^{6}	4.01×10^{-13}
400	4.10×10^{11}	3.48×10^{8}	2.68×10^{10}	6.78×10^{11}	3.69×10^{7}	2.50×10^{-9}	6.59×10^{10}	1.18×10^{8}	5.22×10^{7}	7.69×10^{-7}

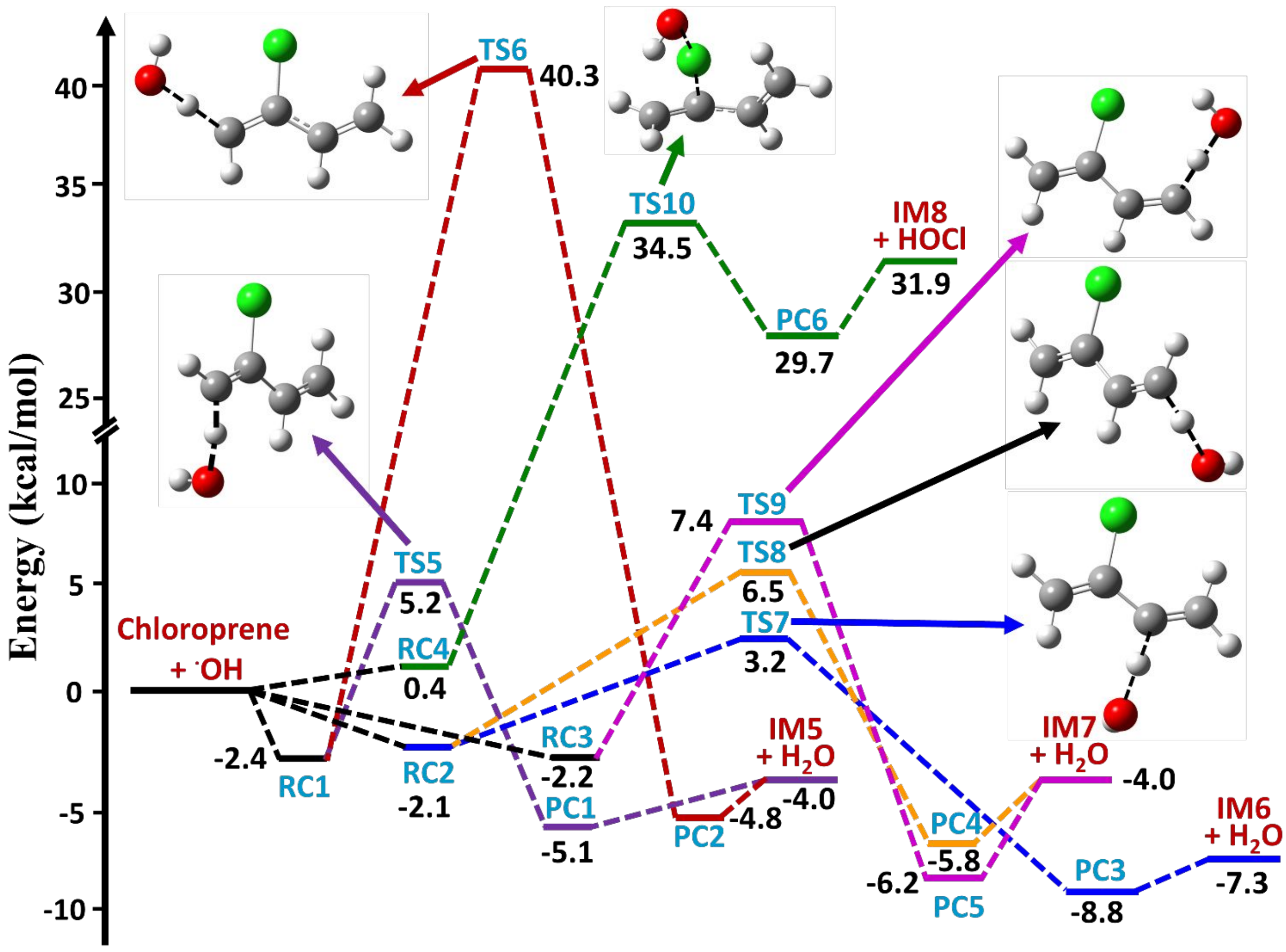

Figure S1. ZPE-corrected CCSD(T)/aug-cc-pVTZ//M06-2X/aug-cc-pVTZ level calculated potential energy profiles for the various abstraction paths involved in the chloroprene + •OH reaction, leading to the formation of their respective C-centered chloroprene radicals + H_2O/HOCl as products. The symbols RCs, TSs, PCs and IMs represent pre-reactive complexes, transition states, post-reactive complexes and products, respectively.

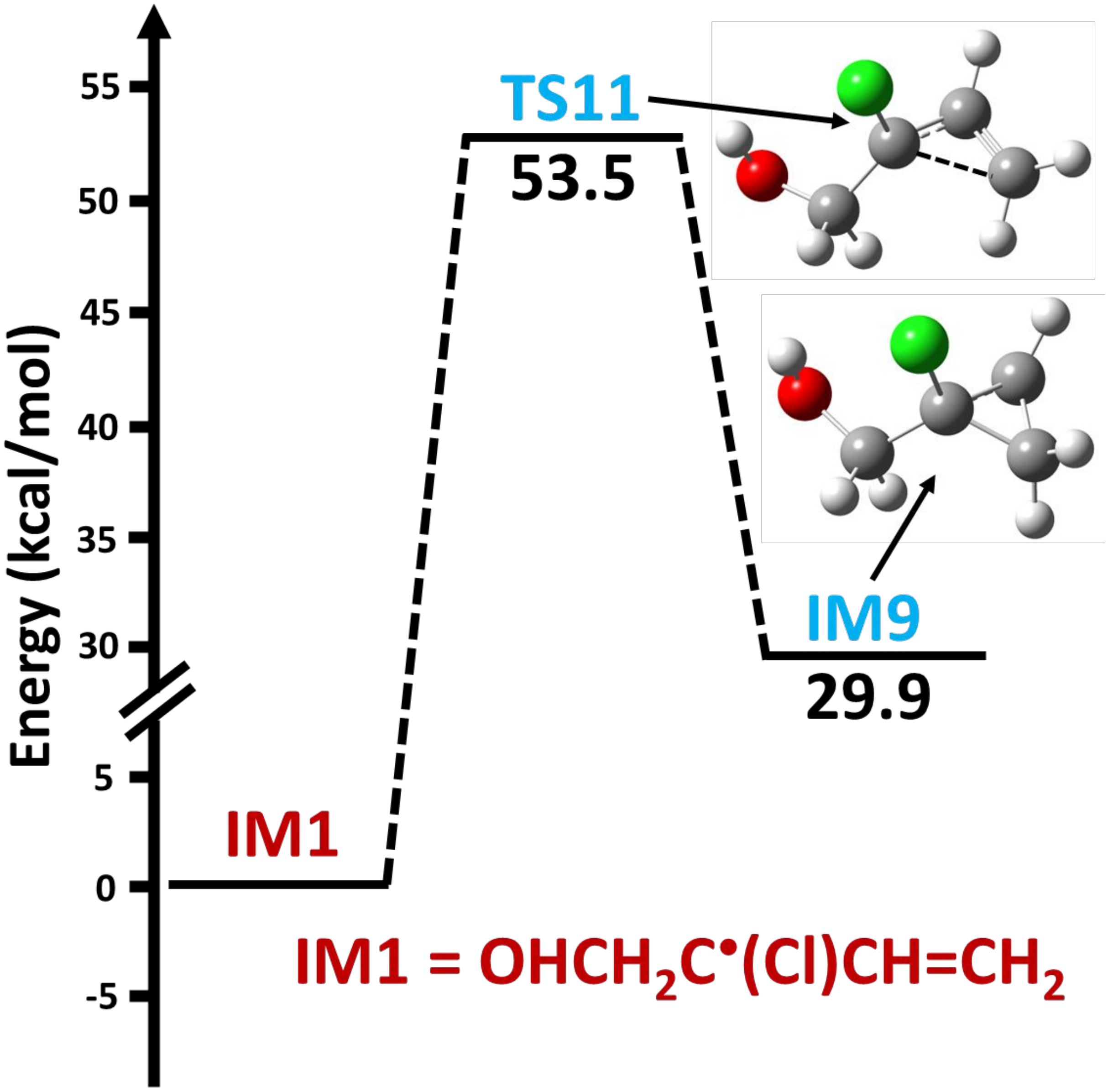

Figure S2. ZPE corrected CCSD(T)/aug-cc-pVTZ//M06-2X/aug-cc-pVTZ level calculated PES profile for the cyclic isomerization reaction of the C-centered chloroprene-OH radical (IM1). The symbols IM1 = $HOCH_2C^{\bullet}(Cl)CH{=}CH_2$; IM9 = cyc-$OHCH_2C(Cl)C^{\bullet}HCH_2$ and TS indicates the transition state.

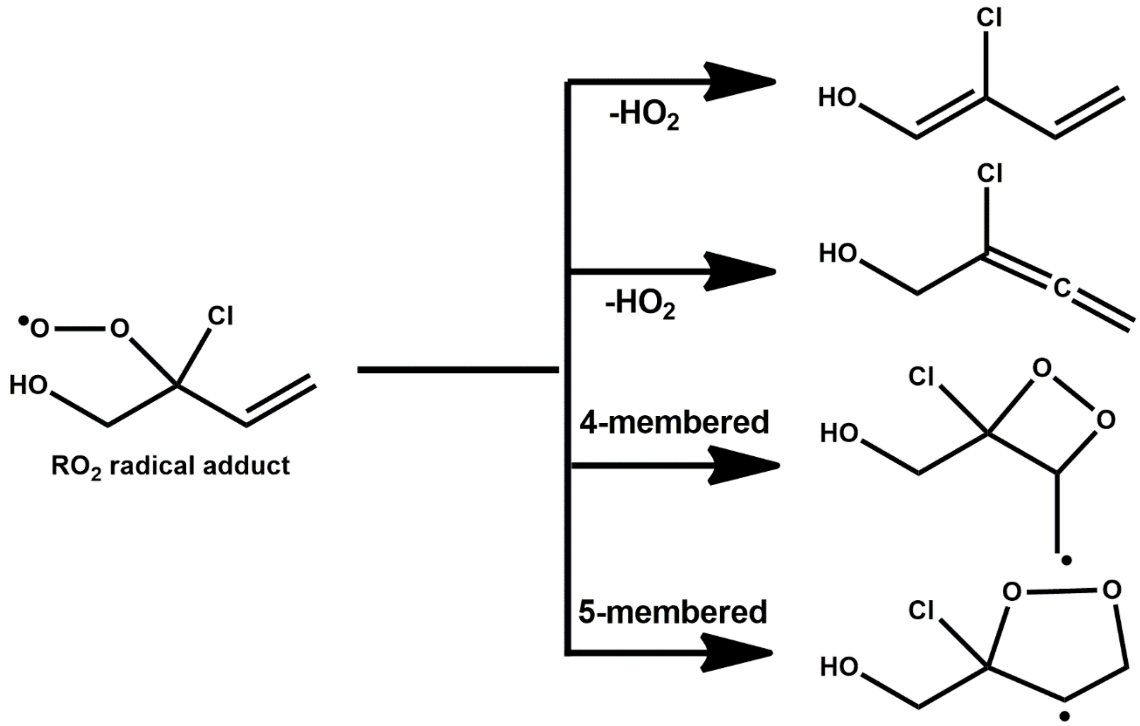

Figure S3. Direct HO_2 elimination and cyclization reaction pathways of the RO_2 radical adduct formed from the reaction of IM1 + O_2.

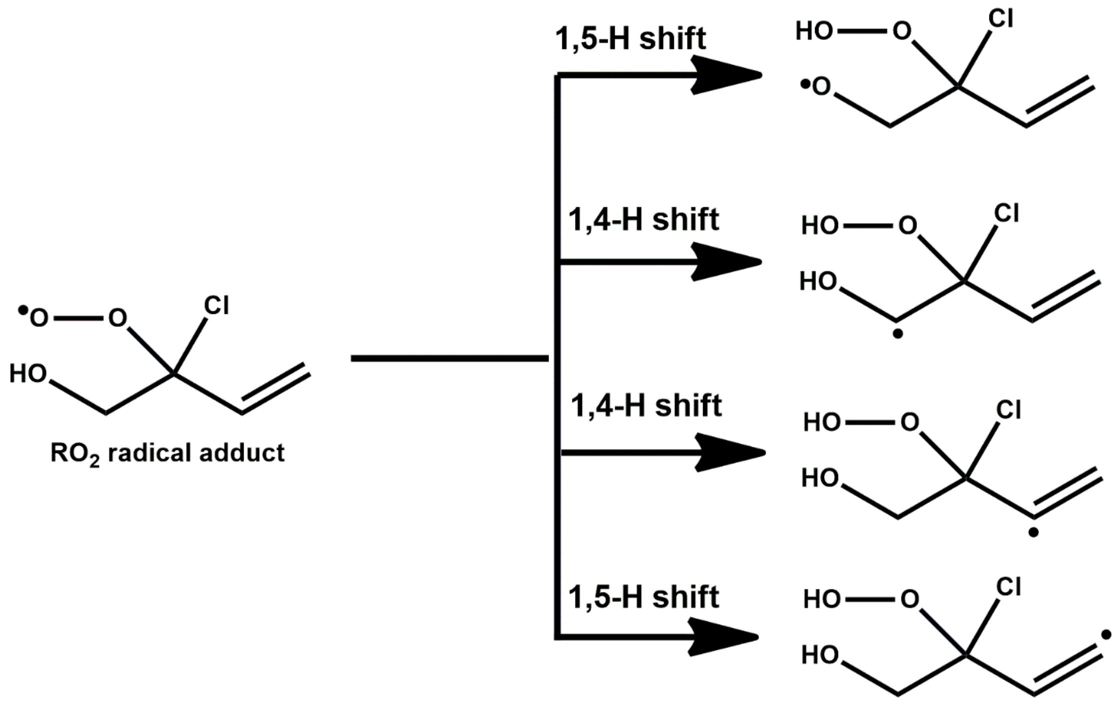

Figure S4. Various possible H-atom shift reactions of the RO_2 radical adduct formed from the reaction of IM1 + O_2.

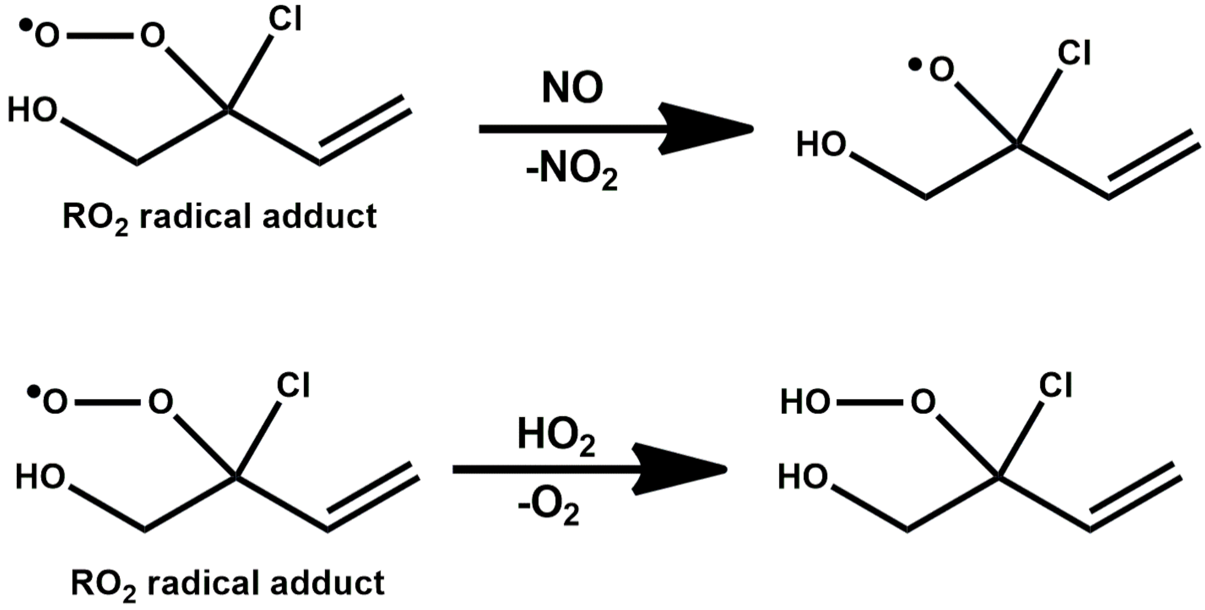

Figure S5. Bimolecular reactions of the RO_2 radical adduct with NO and HO_2 radicals, leading to the formation of the corresponding alkoxy radical + NO_2 and hydroperoxide + O_2, respectively.

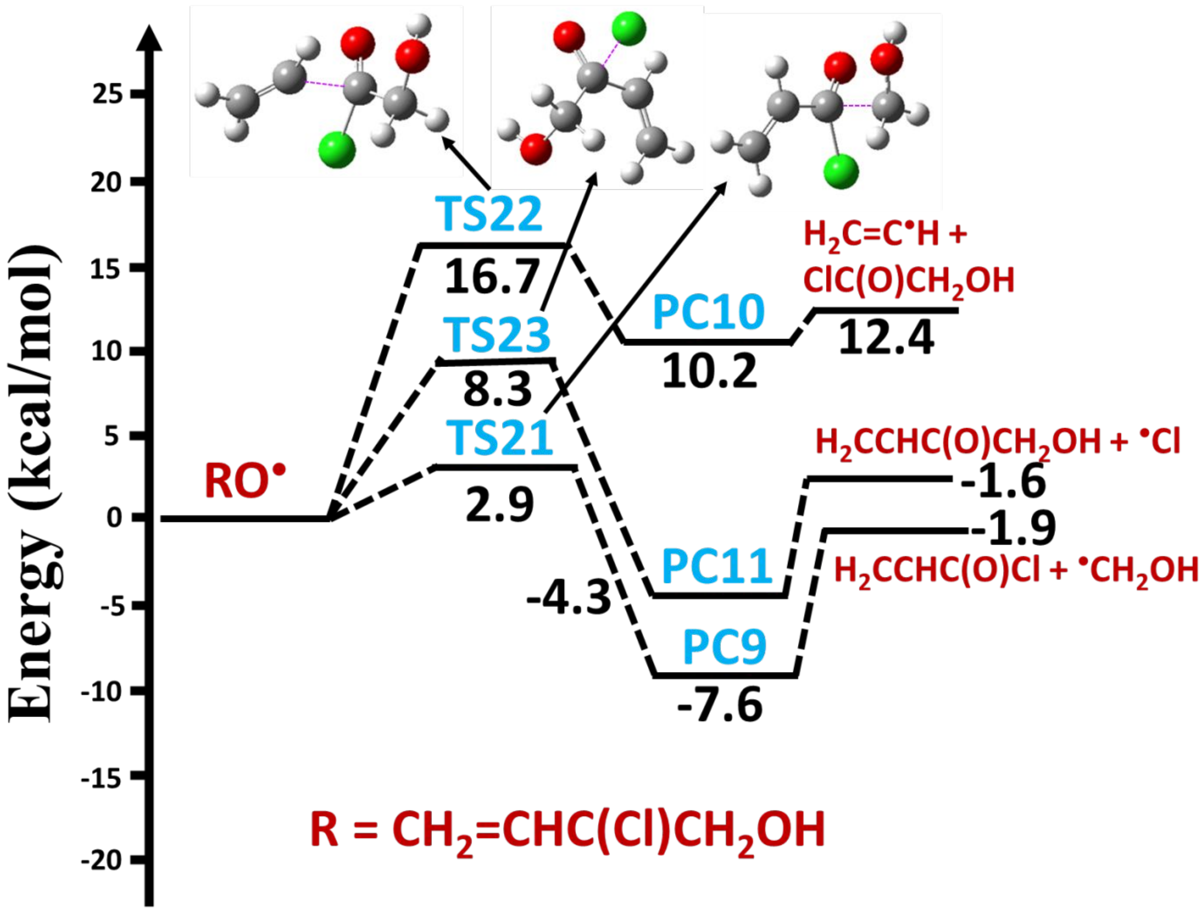

Figure S6: The ZPE-corrected CCSD(T)//M06-2X level calculated PES profiles for the decomposition of RO radical ($H_2C{=}CHC(Cl)(O)CH_2OH$) to form various products.

Section S1—Abstraction pathways: Various possible H-atom abstraction channels proceed to form stable pre-reactive complexes from the starting CP + •OH reactants, which then lead to their respective transition states (TS5, TS6, TS7, TS8 and TS9) with barrier heights of 5.2, 40.3, 3.2, 6.5 and 7.4 kcal mol^{-1} above the starting reactants (see Figure S2). The barrier height for H-atom abstraction from the C_1-atom which points away from the adjacent chlorine atom via TS5, was found to be 35.1 kcal mol^{-1} lower compared to the value for abstraction of the other H-atom that is attached to the same carbon, but which is oriented towards the chlorine atom via TS6. The significantly larger barrier for TS6 may be due to the presence of a hydrogen bond between the H-atom and adjacent Cl atom with a bond length of 2.81 Å, which was observed in free CP. Abstraction of an H-atom by •OH would break this hydrogen bond which would facilitate occurrence of this reaction. This type of interaction is absent for the H-atom that is oriented away from the Cl-atom (see structures TS5 and TS6 in Figure S2) and hence, the TS5 barrier is found to be smaller. The structure of TS7 suggests that •OH abstracts the H-atom from the -CH moiety of

CP. The structures of TS8 and TS9 clearly suggest abstraction of H-atoms from the C_4-atom. In all these abstraction transition states, bond cleavage and formation are shown with dotted lines (see Figure S2). The corresponding post-reactive complexes (PC1-PC5) were formed from these transition states at -5.1, -4.8, -8.8, -5.8 and -6.2 kcal mol^{-1} below the starting reactants. These then go on to form the corresponding C-centered CP radicals (IM5 ($HC^{\bullet}=C(Cl)CH=CH_2$); IM6 ($H_2C=C(Cl)C^{\bullet}=CH_2$); and IM7 ($H_2C=C(Cl)CH=^{\bullet}CH$)) + H_2O on the PES at -4.0, -7.3, and -4.0 kcal mol^{-1} (see Figure S2). We found that abstraction of a Cl-atom by $^{\bullet}OH$ proceeds via RC4, TS10, PC6 and IM8 ($H_2C=C^{\bullet}-CH=CH_2$) + HOCl as stationary points on the PES. The barrier height for TS10 was found to be 34.5 kcal mol^{-1} above the CP + $^{\bullet}OH$ reactants. The H- and Cl-atom abstraction barrier heights via TS6 and TS10 were found to be significantly high and hence they were not feasible under normal atmospheric conditions.
